# Single nucleotide polymorphisms reveal a genetic cline across the north‐east Atlantic and enable powerful population assignment in the European lobster

**DOI:** 10.1111/eva.12849

**Published:** 2019-08-07

**Authors:** Tom L. Jenkins, Charlie D. Ellis, Alexandros Triantafyllidis, Jamie R. Stevens

**Affiliations:** ^1^ Department of Biosciences, College of Life and Environmental Sciences University of Exeter Exeter UK; ^2^ National Lobster Hatchery South Quay Padstow UK; ^3^ School of Biology Aristotle University of Thessaloniki Thessaloniki Greece

**Keywords:** assignment, connectivity, fisheries, Fluidigm EP1, genetic structure, lobster, RAD sequencing, single nucleotide polymorphism (SNP)

## Abstract

Resolving stock structure is crucial for fisheries conservation to ensure that the spatial implementation of management is commensurate with that of biological population units. To address this in the economically important European lobster (*Homarus gammarus*), genetic structure was explored across the species' range using a small panel of single nucleotide polymorphisms (SNPs) previously isolated from restriction‐site‐associated DNA sequencing; these SNPs were selected to maximize differentiation at a range of both broad and fine scales. After quality control and filtering, 1,278 lobsters from 38 sampling sites were genotyped at 79 SNPs. The results revealed a pronounced phylogeographic break between the Atlantic and Mediterranean basins, while structure within the Mediterranean was also apparent, partitioned between lobsters from the central Mediterranean and the Aegean Sea. In addition, a genetic cline across the north‐east Atlantic was revealed using both putatively neutral and outlier SNPs, but the precise driver(s) of this clinal pattern—isolation by distance, secondary contact, selection across an environmental gradient, or a combination of these factors—remains undetermined. Putatively neutral markers differentiated lobsters from Oosterschelde, an estuary on the Dutch coast, a finding likely explained by past bottlenecks and limited gene flow with adjacent North Sea populations. Building on the findings of our spatial genetic analysis, we were able to test the accuracy of assigning lobsters at various spatial scales, including to basin of origin (Atlantic or Mediterranean), region of origin and sampling location. The predictive model assembled using 79 SNPs correctly assigned 99.7% of lobsters not used to build the model to their basin of origin, but accuracy decreased to region of origin and again to sampling location. These results are of direct relevance to managers of lobster fisheries and hatcheries, and provide the basis for a genetic tool for tracing the origin of European lobsters in the food supply chain.

## 1 INTRODUCTION

Identifying distinct genetic diversity among populations and delineating biologically accurate management units are key objectives of conservation biology and fisheries management (Funk, McKay, Hohenlohe, & Allendorf, 2012; Palsbøll, Bérubé, & Allendorf, 2007). For managing fisheries, it is important to identify stock structure and connectivity to ensure that the spatial implementation of management is commensurate with that of biological population units (Reiss, Hoarau, Dickey‐Collas, & Wolff, 2009), and to pinpoint populations that may contribute colonizers to overfished or depleted stocks (Da Silva, Appleyard, & Upston, 2015). Moreover, this information is equally important for hatchery stocking programmes so that managers can ensure that the juveniles they release are compatible with the target population and the area being stocked (Ward, 2006). For example, supplementing a focal population with genetically incompatible sources (e.g. individuals adapted to a very different environment) may lead to undesired negative consequences, such as mortality and outbreeding depression (Frankham et al., 2011).

Delineating subtle population structure using genome‐wide single nucleotide polymorphisms (SNPs), isolated from restriction‐site‐associated DNA sequencing (RADseq) (Baird et al., 2008) and other genomics techniques (Campbell, Brunet, Dupuis, & Sperling, 2018), has become commonplace in population genetics and molecular ecology (Allendorf, Hohenlohe, & Luikart, 2010; Andrews, Good, Miller, Luikart, & Hohenlohe, 2016; Davey et al., 2011). Such approaches have enabled researchers to resolve fine‐scale population structure in a range of marine species, including American lobster (Benestan et al., 2015), great scallop (Vendrami et al., 2017), peacock wrasse (Carreras et al., 2017) and emperor penguin (Younger et al., 2017). Conversely, SNPs have also confirmed the existence of no or only weak population structure in some species across a variety of spatial scales (Everett et al., 2016; Pérez‐Portela et al., 2018), an equally important finding for marine management as it implies genetic connectivity and/or large effective population sizes across the geographical area studied. Genomics has also contributed to the discovery of outlier loci (i.e. loci with high *F*_st_ relative to neutral expectations), which are markers potentially under the influence of selection (Lotterhos & Whitlock, 2015). From a conservation perspective, these outlier markers have the potential to aid the delineation of conservation units by identifying adaptive diversity in protected or exploited species (Barbosa et al., 2018; Flanagan, Forester, Latch, Aitken, & Hoban, 2017; Funk et al., 2012). Moreover, these markers often have greater power to differentiate populations, which offers promising applications for detecting immigrants via assignment approaches (Gagnaire et al., 2015). Indeed, the incorporation of gene‐associated markers in assignment analyses has already proven to be incredibly useful in fisheries management, where these markers have been developed as tools to help tackle illegal fishing (Martinsohn & Ogden, 2009; Nielsen et al., 2012).

The ability to isolate informative SNPs (i.e. SNPs that show the greatest allele frequency variation between putative populations) has permitted the development of small panels of SNP markers which capture differentiation at the spatial scales of interest (e.g. Gilbey et al., 2016; Jenkins, Ellis, & Stevens, 2018; Meek et al., 2016; Nielsen et al., 2012; Villacorta‐Rath et al., 2016). Although useful for detecting subtle differentiation that can enhance analyses of population structure and assignment, the most differentiated loci (i.e. outlier loci) can have complex evolutionary histories of divergence, which may not always be representative of neutral genome‐wide patterns (Gagnaire et al., 2015). As a result, interpreting patterns of dispersal and gene flow based on these loci can be challenging unless the evolutionary mechanisms that gave rise to the outlier loci are identified (Gagnaire et al., 2015). Nevertheless, identifying neutral markers and omitting outlier loci from the population genetic analysis can also be employed to provide insights into processes that influence gene flow and drift, such as allopatric divergence and changes in effective population sizes.

The European lobster (*Homarus gammarus*) is a large decapod crustacean usually found hiding in crevices within hard substrates from the low‐tide mark to 150 m, but typically at depths not exceeding 50 m. The current range of *H. gammarus* extends over most of the north‐east Atlantic, from northern Norway to northern Morocco (but not the Baltic Sea), and includes parts of the Mediterranean and the western Black Sea where they are considerably rarer (Spanier et al., 2015). The high market value of *H. gammarus*, one of the UK's most valuable export species by weight (£14.06 kg^−1^ on average in 2017—more than triple that of cod; Seafish, 2018), makes it a prized seafood product; thus, its fisheries are of great importance to the local and regional economies they support. However, recent and historical overexploitation has led to profound stock declines, with several regions (e.g. Scandinavia, the Mediterranean and the western Black Sea) experiencing severe stock collapses, from which recovery has been slow or stagnant (Agnalt, Kristiansen, & Jørstad, 2007; Kleiven, Olsen, & Vølstad, 2012; Spanier et al., 2015). This has led to the rearing of *H. gammarus* larvae in lobster hatcheries to produce juveniles which can be released into the wild to supplement or rebuild wild stocks (Agnalt et al., 2004; Bannister & Addison, 1998; Ellis et al., 2015).

Previous genetic studies based on allozyme and mitochondrial DNA (mtDNA) restriction fragment length polymorphism (RFLP) markers have found that lobsters from northern Norway, Oosterschelde (Netherlands) and the Mediterranean are genetically differentiated from each other and all other samples analysed (Jørstad, Faresteit, Kelly, & Triantaphyllidis, 2005; Triantafyllidis et al., 2005). Using 14 microsatellites, Ellis, Hodgson, Daniels, Collins, and Griffiths (2017) suggested lobsters from the Skagerrak region—a strait located between the Jutland and Scandinavian peninsulas which connects the North Sea to the Kattegat and the Baltic Sea—may be genetically distinct. However, the two Scandinavian sites used in this microsatellite‐based study were genotyped by a different laboratory from the main group of samples analysed, and since accurate cross‐calibration of microsatellite profiles between laboratories is notoriously difficult (Ellis et al., 2011), the role of differences in locus‐calling between laboratories could not be ruled out as a causal factor of the differentiation reported. Elsewhere in the north‐east Atlantic, virtually no genetic differentiation between samples of European lobster has been found using microsatellite markers (Ellis et al., 2017; Huserbraten et al., 2013; Watson, McKeown, Coscia, Wootton, & Ironside, 2016). Nevertheless, it is uncertain whether the apparent lack of population structure across much of the European lobster's range reflects genuine panmixia via widespread dispersal (or large effective population sizes), or simply limitations in the analytical power provided by small arrays of microsatellites to detect weak spatial structuring.

The first goal of this study, therefore, was to explore broad‐ and fine‐scale population structure across the range of European lobster using a panel of informative SNP markers isolated from RADseq data, and to compare results with previous studies that employed traditional molecular markers. The second goal of this study was to assess the accuracy of this SNP panel to assign individual lobsters back to their place of origin at different spatial scales, including to broad‐scale ocean basin (Atlantic or Mediterranean), intermediate‐scale region and fine‐scale location (sampling location). Finally, we discuss the applications of these results to inform the management, supplementation and conservation of European lobster populations.

## 2 MATERIALS AND METHODS

### 2.1 Sampling and DNA extraction

**Figure 1:**
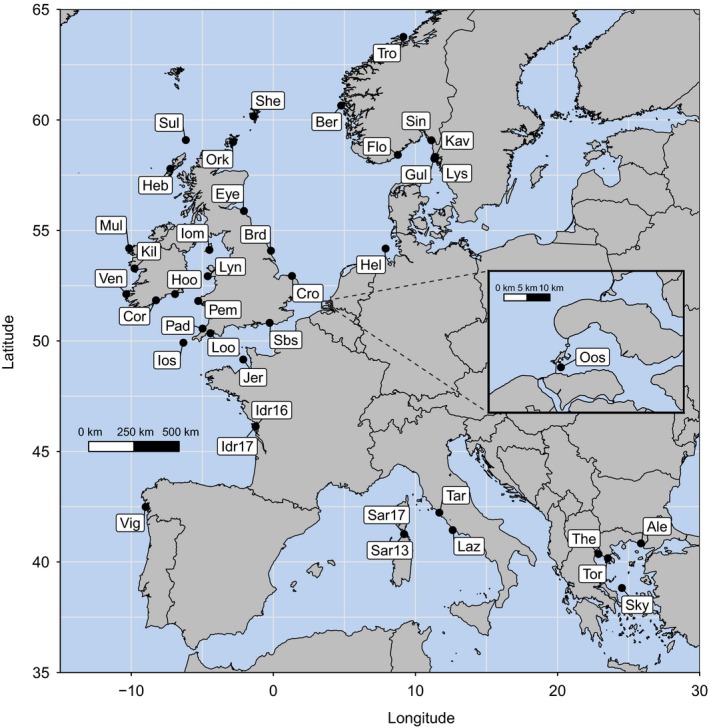
Map of the study area showing the locations of the sites sampled across the north‐east Atlantic and the Mediterranean. See Table 1 for detailed information about sites and sampling

Samples of adult European lobsters were collected from 38 sites (together with two temporal samples from Île de Ré and Sardinia; Table 1, Figure 1), covering most of the contemporary geographical range of *H.* *gammarus*. The majority of sites were sampled in 2016–2018; however, due to the rarity and difficulty of obtaining Mediterranean samples, some DNA samples analysed in previous studies (Ellis et al., 2017; Triantafyllidis et al., 2005) were also utilized in this study. In addition, several Scandinavian samples were collected in 2007 and 2009 (provided by Carl André, University of Gothenburg). Nondestructive tissue samples were obtained by excising a 1‐ to 2‐cm distal section from one or two pleopods, although tissue samples from a few sites were composed of pereiopods, antennae or the uropod (v‐notches; Table 1). All samples were placed in 95%–100% ethanol and stored at 4°C for long‐term preservation. Genomic DNA was extracted from all tissue types using a modified salting‐out protocol (Jenkins et al., 2018). The concentration and purity of all DNA extractions were quantified by spectrophotometry using a NanoDrop 1000.

**Table 1:** Summary of sampling information and heterozygosity using 79 single nucleotide polymorphisms

Country	Site	Code	*N*	Lat	Lon	Tissue type	Year	*H*_o_	*H*_e_
Britain	Bridlington	Brd	36	54.07	−0.17	Pleopods	2017	0.37	0.36
	Cromer	Cro	35	52.94	1.31	Pleopods	2016	0.37	0.37
	Eyemouth	Eye	32	55.88	−2.07	Pleopods	2017	0.38	0.37
	Outer Hebrides	Heb	36	57.79	−7.25	Pleopods	2017	0.39	0.38
	Isle of Man	Iom	35	54.12	−4.50	Pleopods	2016	0.39	0.38
	Isles of Scilly	Ios	36	49.92	−6.33	Pleopods	2016	0.39	0.38
	Looe Harbour	Loo	36	50.35	−4.44	Pleopods	2016	0.39	0.37
	Llyn Peninsula	Lyn	34	52.93	−4.62	Pleopods	2017	0.40	0.38
	Orkney	Ork	36	59.00	−2.83	Pleopods	2017	0.36	0.36
	Padstow	Pad	36	50.56	−4.98	Pleopods	2017	0.37	0.37
	Pembrokeshire	Pem	36	51.81	−5.29	Pleopods	2016	0.38	0.37
	Shetland	She	36	60.17	−1.40	Pleopods	2017	0.37	0.36
	Shoreham‐By‐Sea	Sbs	36	50.82	−0.26	Pleopods	2016	0.38	0.37
	Sula Sgeir	Sul	36	59.09	−6.16	Pleopods	2017	0.36	0.37
Channel Islands	Jersey	Jer	36	49.16	−2.12	Pleopods	2016	0.38	0.37
France	Île de Ré, La Rochelle	Idr16	32	46.13	−1.25	V‐notches	2016	0.39	0.38
		Idr17	29	46.13	−1.25	V‐notches	2017	0.39	0.38
Germany	Helgoland	Hel	35	54.18	7.90	Pleopods	2017	0.35	0.34
Greece	Alexandroupoli	Ale	28	40.84	25.87	DNA	1999–2001	0.32	0.33
	Skyros	Sky	37	38.82	24.53	DNA	1999–2001	0.33	0.33
	Thermaikos Bay	The	36	40.36	22.88	DNA	1999–2001	0.33	0.33
	Toronaios Bay	Tor	37	40.17	23.54	DNA	1999–2001	0.33	0.33
Ireland	Cork	Cor	32	51.84	−8.26	Pleopods	2016	0.38	0.38
	Hook Peninsula	Hoo	36	52.12	−6.92	V‐notches	2016	0.39	0.38
	Kilkieran Bay	Kil	35	53.28	−9.77	Pleopods	2016	0.38	0.37
	Mullet Peninsula	Mul	36	54.19	−10.15	V‐notches	2016	0.37	0.38
	Ventry	Ven	36	52.12	−10.35	V‐notches	2016	0.39	0.37
Italy	Lazio	Laz	5	41.44	12.62	Antennae	2013	0.38	0.31
	Tarquinia, Lazio	Tar	5	42.23	11.68	Antennae	2013	0.42	0.32
	Sardinia	Sar13	7	41.26	9.20	Antennae	2013	0.32	0.30
		Sar17	15	41.26	9.20	Pleopods	2017	0.34	0.33
Netherlands	Oosterschelde	Oos	40	51.61	3.70	Pleopods	2017	0.32	0.33
Norway	Bergen	Ber	33	60.65	4.77	Pleopods	2018	0.36	0.35
	Flødevigen	Flo	36	58.42	8.76	Pleopods	2016	0.35	0.34
	Singlefjord	Sin	36	59.08	11.12	Pleopods	2009	0.35	0.35
	Trondheim	Tro	17	63.76	9.15	Pleopods	2018	0.33	0.35
Spain	Vigo	Vig	36	42.49	−8.99	Pleopods	2017	0.39	0.39
Sweden	Gullmarfjord	Gul	35	58.25	11.33	Pereiopods	2009	0.38	0.36
	Kavra	Kav	36	58.33	11.37	Pereiopods	2007	0.37	0.36
	Lysekil	Lys	36	58.26	11.37	Pleopods	2017	0.34	0.34

Abbreviations: *H*_e_, expected heterozygosity; *H*_o_, observed heterozygosity; *N*, number of individuals genotyped.

### 2.2 SNP isolation and genotyping

Single nucleotide polymorphism genotyping was carried out on a Fluidigm EP1 system using the 96 SNPs isolated by Jenkins et al., (2018). In brief, for SNP discovery, RAD sequencing using the *SbfI* restriction enzyme was performed on a subset of 55 lobsters from 27 geographically separate sampling sites (Figure S1A), encompassing much of the present‐day range of *H. gammarus*. Two SNP data sets were generated after bioinformatic analysis with Stacks v1.45 (Catchen, Hohenlohe, Bassham, Amores, & Cresko, 2013). The first SNP data set comprised all 55 individuals from 27 sampling sites genotyped at 7,022 SNPs, and initial analysis with discriminant analysis of principal components (DAPC; Jombart et al., 2010) clustered these individuals into three main groups: the Atlantic, Skagerrak and the Mediterranean (Figure S1B). All 55 individuals were then organized into these three putative groups, and each SNP was ranked using *F*‐statistics with the aim of identifying SNPs that were most informative for maximizing genetic differentiation between these groups. Global *F*_st_ (Weir & Cockerham, 1984) between these three groups was 0.018, calculated using the *diffCalc* function from diveRsity v1.9.90 (Keenan, Mcginnity, Cross, Crozier, & Prodohl, 2013) implemented in R (R Core Team, 2018). The second SNP data set comprised 40 individuals that originated from only the Atlantic (excluding Mediterranean and Skagerrak samples; Table S1). To generate this data set, a population map that organized the remaining Atlantic samples into geographical regions (Table S1) was submitted to the *populations* program in Stacks. Subsequently, this SNP data set comprised 40 individuals from nine putative populations genotyped at 4,377 SNPs. Each SNP was also ranked using *F*‐statistics with the aim of identifying SNPs that are most informative for maximizing fine‐scale differentiation between lobsters originating from different regions in the Atlantic, which have to‐date been found to be genetically homogeneous; global *F*_st_ for this data set was 0.002.

Due to the requirements of the Fluidigm EP1 system (i.e. dependency on 96‐well plates), 96 SNPs were used to compose the panel. After ranking SNPs in both data sets by *G*″_st_ (Meirmans & Hedrick, 2011; other differentiation measures produced similar results), and after filtering SNPs that were ineligible for primer design and synthesis, 21 SNPs (out of 7,022 SNPs) were selected to capture differentiation between Atlantic, Skagerrak and Mediterranean lobsters (Figure S1C), and 75 SNPs (out of 4,377 SNPs) were selected to capture within‐Atlantic differentiation (Figure S1D). Fluidigm SNP assays and DNA samples were run on a 96.96 Dynamic Array integrated fluidic circuit, and genotypes were called using the Fluidigm SNP Genotyping Analysis software. Specific target amplification (STA) was conducted prior to genotyping because it increases the copy numbers of the desired sequence containing the SNP (i.e. the RAD‐tag), which can improve genotyping call rates and accuracy, particularly for heterozygous samples (Bhat, Polanowski, Double, Jarman, & Emslie, 2012).

### 2.3 Quality control and filtering

Individuals and SNP loci with more than 20% missing genotypes were removed from the data set using the *missingno* function from poppr v2.8.0 (Kamvar, Tabima, & Grünwald, 2014). Deviations from Hardy–Weinberg equilibrium (HWE) were tested using the *hw.test* function from pegas v0.11 (Paradis, 2010) using the exact test based on Monte Carlo permutations of alleles (1,000 replicates); loci were considered to be out of HWE if they deviated significantly (*p* < .05) in more than 50% of populations. Although only one target SNP per RAD‐tag was considered to compose the SNP panel in the RADseq study (Jenkins et al., 2018), linkage disequilibrium (LD) was also tested in this study using the *LD2* function from pegas. For both HWE and LD, the Bonferroni correction was used to adjust for multiple comparisons.

### 2.4 Outlier selection tests

Outlier selection tests were conducted on the original RAD data from Jenkins et al., (2018) using three differentiation‐based approaches: Bayescan v2.1 (Foll & Gaggiotti, 2008), OutFLANK v0.2 (Whitlock & Lotterhos, 2015) and PCadapt v4.0.3 (Luu, Bazin, & Blum, 2017). Bayescan is a Bayesian method based on a logistic regression model that attempts to distinguish locus‐specific (alpha) effects of selection from population‐specific (beta) effects of demography; departure from neutrality at a given locus is assumed when the locus‐specific component is required to explain the observed pattern of diversity (Foll & Gaggiotti, 2008). Bayescan was run using default parameters and a prior odds of 10,000, which sets the neutral model as being 10,000 times more likely than the model of selection to minimize the risk of false positives. OutFLANK calculates a likelihood on a trimmed distribution of *F*_st_ values to infer the distribution of *F*_st_ for neutral markers; it was executed using default parameters. PCadapt uses principal component analysis (PCA) to detect loci under selection and assumes that markers excessively related to population structure are candidates for local adaptation. For all selection tests, an alpha of 0.05 was used, and loci that were identified as an outlier in two or more tests were considered outlier SNPs.

### 2.5 Genetic differentiation and population structure

Analyses of genetic differentiation between sampling sites were conducted on the SNP panel data by calculating pairwise values of *F*_st_ (Weir & Cockerham, 1984) and *D* (Jost, 2008) using the *diffCalc* function from diveRsity. Heat maps of each statistic were visualized in R, and significance was assessed by calculating bias‐corrected 95% confidence intervals (1,000 replicates) and testing whether values were significantly different from zero. In addition, to explore patterns of isolation by distance (IBD), Mantel tests were conducted on genetic distances (*F*_st_) and geographical distances (km) using the *mantel.rtest* function from the R package ade4 v1.7.11 (Dray & Dufour, 2007). The geographical distance matrices were created by calculating least‐cost distances via seas (avoiding landmasses) between sampling sites using the *lc.dist* function from the R package marmap v1.0 (Pante & Simon‐Bouhet, 2013). Outlier SNPs were omitted for this analysis, and significance of the Mantel tests was assessed using 1,000 permutations.

Population structure was explored using three different approaches. First, DAPC was run using the *dapc* function from the R package adegenet v2.1.1 (Jombart & Ahmed, 2011). DAPC attempts to summarize genetic differentiation between groups (between sampling sites, in this context), while overlooking variation within groups (Jombart et al., 2010). DAPC does not assume a population genetics model; instead, it transforms the data using PCA and then performs discriminant analysis on the number of principal components retained. Cross‐validation using the *xvalDapc* function from adegenet was used to choose the optimal number of principal components to retain. Second, the program snapclust (Beugin, Gayet, Pontier, Devillard, & Jombart, 2018) was implemented in adegenet. This program uses maximum‐likelihood estimations based on the expectation–maximization algorithm to investigate genetic clustering and admixture, assuming HWE and independence of loci (linkage equilibrium). The number of clusters best describing the pattern of differentiation was explored by examining the DAPC results and by computing goodness‐of‐fit statistics. To visualize the genetic clusters geographically, individual membership proportions to each *K* cluster were averaged for each sampling site and the data were plotted as pie charts on a map. Lastly, STRUCTURE v2.3.4 (Pritchard, Stephens, & Donnelly, 2000), a Bayesian clustering algorithm, was run in parallel using the program StrAuto v1.0 (Chhatre & Emerson, 2017). STRUCTURE attempts to estimate the number of ancestral populations (*K*) from multilocus allele frequencies, with the assumption that loci are in HWE and linkage equilibrium. STRUCTURE was executed using the admixture model, with 10^5^ MCMC repetitions and a burn‐in of 10^5^. The locprior option was selected, meaning sampling locations were used as a priori information; all other parameters were set to default values. To statistically compare different values of *K*, the mean value of L (*K*) (Pritchard et al., 2000) and the delta *K* (Evanno, Regnaut, & Goudet, 2005) statistics were examined in the R package pophelper v2.2.5.1 (Francis, 2017). Replicate runs were aligned and merged with CLUMPP v1.1.2 (Jakobsson & Rosenberg, 2007) using a wrapper script in pophelper, and R was used to visualize the results.

### 2.6 Individual assignment

The accuracy of assigning individuals back to their basin (Atlantic or Mediterranean), region and sampling location of origin was assessed using the R package assignPOP v1.1.4 (Chen et al., 2018). assignPOP uses a cross‐validation procedure followed by PCA to evaluate assignment accuracy and membership probabilities. First, the data set is partitioned into training (baseline) and test (holdout) data sets using a resampling cross‐validation procedure, with the user specifying the number or proportion of individuals from each source “population” (i.e. Atlantic or Mediterranean in the basin analysis) to be used in the training data set. This approach of creating randomly selected, independent training and test data sets avoids introducing high‐grading bias (Anderson, 2010). Second, the features of the training data sets (i.e. the genotypes) are reduced in dimensionality using PCA, the output of which are used to build predictive models from user‐chosen classification machine‐learning functions (Chen et al., 2018). Finally, these models are then used to estimate membership probabilities of test individuals and assign them to a source population, while also evaluating the baseline data and conducting assignment tests on individuals for which the origin is unknown (Chen et al., 2018).

For assigning individuals to their basin of origin, before dividing the data set into baseline and test data sets, two individuals from each Mediterranean site (16 individuals in total) were randomly selected in R to compose a file representing “unknown” individuals, whereby the basin of origin was considered to be unknown. Due to the potential bias of unequal sample size in assignment analyses (Wang, 2017), 250 individuals from the Atlantic basin were randomly selected in R to compose this source population, with 154 individuals composing the Mediterranean basin. The remaining individuals from the Atlantic (858 individuals) were added to the “unknown” file (874 individuals in total).

A Monte Carlo cross‐validation procedure was used to group individuals into baseline and test data sets using the function *assign.MC* from assignPOP. Resampling was repeated 100 times for each combination of training individuals and loci. The proportion of individuals from each source population randomly allocated to the baseline data set was set to 0.5, 0.7 and 0.9. Lastly, the support vector machine (SVM) classification function was used to build predictive models; after building predictive models based on the baseline data set, the origin of the “unknown” individuals was assessed to further evaluate the performance of the predictive model.

## 3 RESULTS

### 3.1 Genotyping, quality control and outlier SNPs

Five SNP loci (25580, 32362, 41521, 53889 and 65376) did not work consistently on the Fluidigm EP1 system, possibly due to inadequate assay design, poor STA performance or ascertainment bias. One locus (22365) contained 28.3% missing data and two loci (8953 and 21197) deviated significantly from HWE in 22 and 34 sampling sites, respectively; all three loci were subsequently removed from the data set. Two loci (21880 and 22323) exhibited unexpectedly high proportions of observed heterozygosity (0.61 and 0.67, respectively); these loci were removed because they could contain paralogs, as true variants are often considered to have a maximum frequency of 0.50 heterozygous genotypes (Dufresne, Stift, Vergilino, & Mable, 2014). Tests of linkage revealed significant deviation (*p* < .05) from equilibrium between several pairs of loci. In particular, LD was detected between SNPs 15531 and 53935, between SNPs 28357 and 56785, and between SNPs 22740, 33066, 51507, 53052, 53263 and 65064; the extent of LD among these pairs of loci was apparent when visualizing the population allele frequencies for each SNP (Figure S2). Of these loci, 53935, 56785 and 65064 were retained, while the other seven loci were removed. Outlier selection tests revealed that, of the remaining loci, eight loci were classed as outlier SNPs (Table S2), of which the RAD‐tag of one SNP (65064) matched hypothetical proteins on BLASTx, although per identity scores were relatively low (<72.2%). The final filtered data set contained 1,278 individual lobsters from 38 sites (plus two temporal samples) genotyped at 79 biallelic SNP loci. This data set composed 15 SNPs from the original panel selected for analysis of Mediterranean‐Atlantic–Skagerrak differentiation and 64 SNPs selected for analysis of within‐Atlantic differentiation (six and 11 loci were omitted from the 21 and 75 SNPs, respectively, from the original panel).

### 3.2 Genetic differentiation

Global values of *F*_st_ and *D* using all 79 SNPs were 0.051 and 0.010, respectively, and both pairwise differentiation statistics showed comparable patterns between sampling sites (Figure S3). Pairwise values of *F*_st_ and *D* ranged from zero (e.g. Cor‐Hoo) to 0.246 (Oos‐Sar13) and from zero (e.g. Ale‐Sky) to 0.030 (Oos‐Ale), respectively. The highest values for both statistics were between Atlantic sites and Mediterranean sites, of which many values were significantly different from zero. Within the Atlantic, Oosterschelde consistently yielded the highest pairwise values with other Atlantic sites in both statistics (Figure S3). The lowest values tended to be between sites originating from Britain, Ireland and the Channel Islands, although this was also the case between most sites situated close together in other regions (e.g. Greek sites from the Aegean Sea), and between the temporal samples from Île de Ré (2016 and 2017) and Sardinia (2013 and 2017). As a result of their genetic similarity, temporal samples, as well as both sites from Lazio (western Italy), were combined into single samples for the Mantel tests. These tests revealed a strong positive correlation between *F*_st_ and geographical distance using all sites (Figure S4A; *r* ^2^ = 0.87, *p* < .001), although when the Mediterranean samples were removed, this correlation was much weaker (Figure S4B; *r* ^2^ = 0.17, *p* = .060). However, removal of Oosterschelde lobsters from the analysis of Atlantic sites vastly increased the strength and significance of the correlation (Figure S4C; *r* ^2^ = 0.45, *p* < .001). Analysis with only the Mediterranean samples also produced a positive correlation, but this was not significant (Figure S4D, *r* ^2^ = 0.89, *p* = .061).

### 3.3 Population structure and genetic clustering

**Figure 2:**
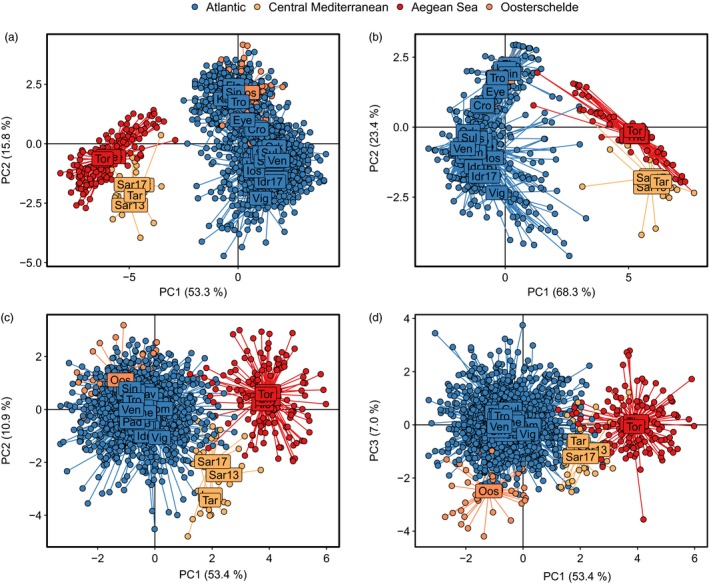
Discriminant analysis of principal components (DAPC): (a) all 79 SNPs; (b) eight outlier SNPs; (c) 71 neutral SNPs (principal components 1 and 2); and (d) 71 neutral SNPs (principal components 1 and 3). For each DAPC, each point represents an individual and colours denote whether the individual originates from the Atlantic (blue), the central Mediterranean (yellow), the Aegean Sea (red) or Oosterschelde (orange)

Analyses of population structure were conducted using all 79 SNPs, and independently using the eight outlier SNPs, and the 71 putatively neutral SNPs (SNPs not identified as outliers, albeit still originally chosen for the panel due to their high differentiation). Global *F*_st_ for the outlier SNP data set was 0.310, while global *F*_st_ for the neutral SNP data set was 0.024. The DAPC using all 79 SNPs showed that lobsters originating from the Atlantic and the Mediterranean were genetically distinct (Figure 2a). There was also evidence for structure within the Mediterranean, partitioned between the central Mediterranean (Sardinia and Lazio samples) and the Aegean Sea (all Greek samples), which was also supported by the pairwise differentiation statistics. Within the Atlantic cluster, there was a clear genetic cline starting from the most southerly site sampled, Vigo (northern Spain), to the most north‐easterly sites sampled in Norway and Sweden. In total, the first and second axes explained 69.1% of the variation in the data set. The outlier SNP data set (Figure 2b) showed very similar patterns to those described using 79 SNPs, but here the first and second axes explained even more of the variation in the data set (91.7%). In contrast, the neutral SNP data set showed a much weaker clinal pattern in the Atlantic, with the first and second axes explaining only 64.3% (Figure 2c). However, compared to the outlier SNPs, neutral SNPs showed stronger separation between the central Mediterranean and the Aegean Sea. Moreover, neutral SNPs still distinguished lobsters originating from the Atlantic and the Mediterranean basins, although the signal was generally weaker. In addition, exploration of the third axis showed that many lobsters from Oosterschelde were markedly differentiated from other Atlantic samples (Figure 2d).

**Figure 3:**
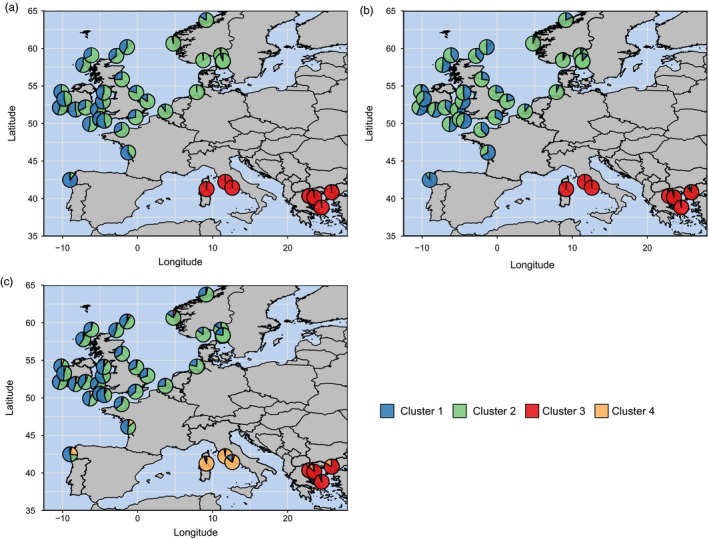
Snapclust results visualized geographically: (a) all 79 SNPs; (b) eight outlier SNPs; and (c) 71 neutral SNPs. Pie chart colours denote the average membership proportions for each sampling site to each *K* cluster

For analyses with snapclust, temporal replicates from Île de Ré and Sardinia were combined into single samples due to their genetic similarity. Analysis of snapclust goodness‐of‐fit statistics revealed support for multiple *K* clusters ranging from 3 to 5 using 79 SNPs (Figure S5), though when the data were visualized, *K* = 3 made most biological sense. Overall, there was virtually no admixture between sites from the Atlantic and sites from the Mediterranean (Figure 3a). Sites in the north‐east Atlantic were predominantly grouped into two clusters (blue and green), both distinct from the Mediterranean (red cluster), and a clinal pattern like the DAPC was apparent. Analysis with outlier SNPs (Figure 3b) showed almost identical patterns to those observed using all 79 SNPs. In contrast, using neutral SNPs (Figure 3c), structure was apparent between sites from the central Mediterranean and sites from the Aegean Sea, which supported the DAPC results. In addition, similarly to the DAPC, neutral SNPs showed a weaker genetic cline in the Atlantic, with some admixture between Atlantic and Mediterranean sites, which appears more prominent in Atlantic sites spatially closer to the Mediterranean.

As a clear genetic cline was detected in the data sets composed of all SNPs and outlier SNPs using DAPC and snapclust, STRUCTURE analysis was deemed to be inappropriate for these two data sets as limitations with the underlying STRUCTURE model can make interpretations extremely challenging when there are clines of genetic variation (Frantz, Cellina, Krier, Schley, & Burke, 2009; Gilbert, 2016; Perez et al., 2018). Nevertheless, as much weaker clinal patterns were detected in the Atlantic using neutral SNPs (Figures 2c and 3c, Figure S4B), hierarchical structure within the Atlantic using only neutral SNPs was analysed with STRUCTURE. This hierarchical analysis revealed *K* = 3 to be informative, and the results supported the differentiation statistics and the DAPC in showing that lobsters from Oosterschelde were genetically differentiated from other Atlantic samples analysed (Figure S6).

### 3.4 Individual assignment

**Figure 4:**
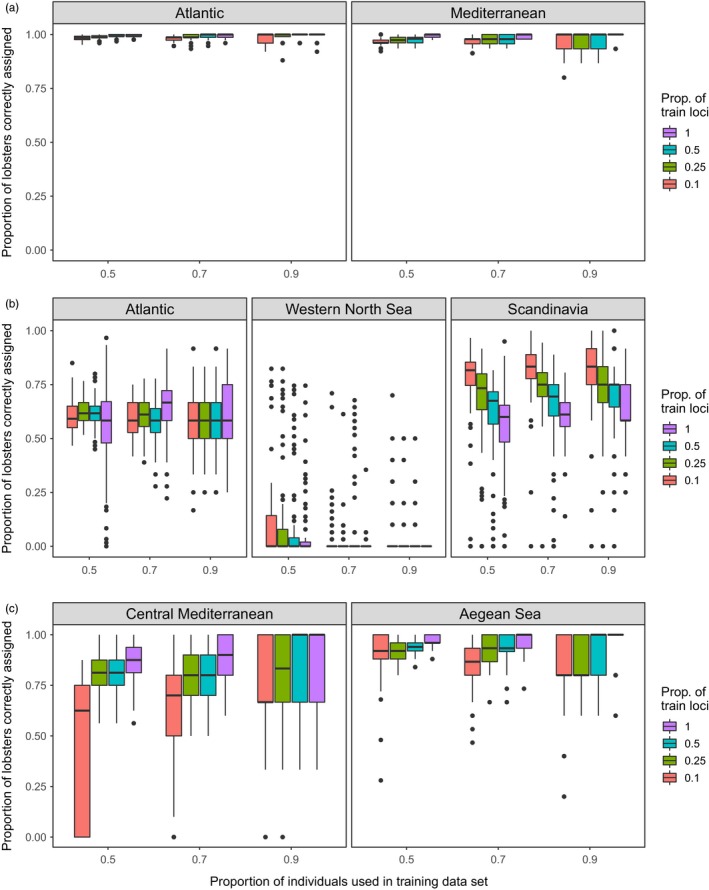
Assignment accuracies estimated via Monte Carlo cross‐validation, with three levels of training (baseline) individuals (50%, 70% and 90% of individuals from each group) crossed by up to four levels of training loci (top 10%, 25%, 50% and all loci) by 100 resampling events: (a) basin of origin analysis; (b) Atlantic region of origin analysis; and (c) Mediterranean region of origin analysis

**Figure 5:**
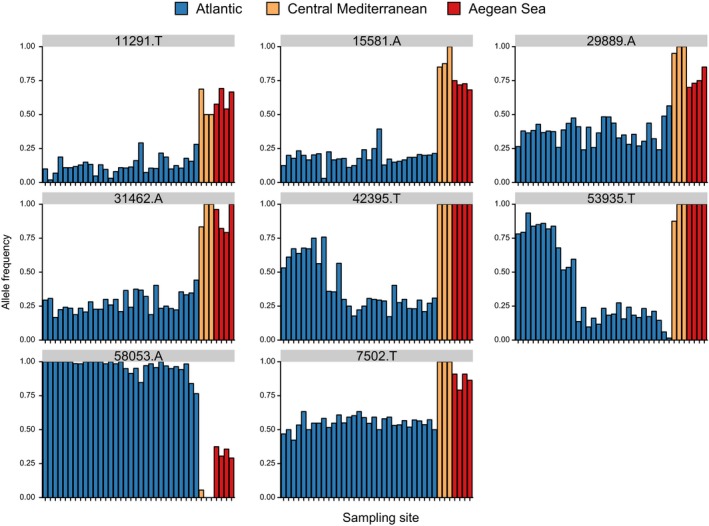
Population allele frequency of one allele for each of the eight top *F*_st_ SNPs identified from the basin of origin assignment analysis. For each SNP, the sampling sites (*x*‐axis) are arranged in the following order: Tro, Ber, Flo, Gul, Kav, Lys, Sin, Hel, Oos, Cro, Brd, Eye, She, Ork, Heb, Sul, Cor, Hoo, Iom, Ios, Jer, Kil, Loo, Lyn, Mul, Pad, Pem, Sbs, Ven, Idr, Vig, Sar, Laz, Tar, Ale, Sky, The and Tor. Colours denote whether the sampling site originates from the Atlantic (blue), the central Mediterranean (yellow) or the Aegean Sea (red)

Assigning individuals to their basin of origin (Atlantic or Mediterranean) using the baseline data was extremely accurate, ranging from 97% to 100% depending on the proportion of individuals used in the training data set and the number of loci used for the assignment tests (Figure 4a). Overall, the proportion of individuals used in the training data set had little effect on assignment accuracy in this analysis. When all 79 SNPs were used, and when a proportion of 0.7 was used for the training data set, the model predicted the basin of origin of Atlantic and Mediterranean test individuals at a mean accuracy of 100% and 99%, respectively. Moreover, the top 10% of high *F*_st_ loci (train.loci = 0.1) correctly assigned on average 98% of Atlantic individuals and 97% of Mediterranean individuals to their basin of origin. The population allele frequency of one allele for each of these top eight SNP loci was visualized (Figure 5); this mostly showed clear allele frequency differences between sites from the Atlantic and sites from the Mediterranean. This model was then tested on the “unknown” data set; the SVM model predicted the correct basin of origin for 99.7% of individuals (871 out of 874 individuals; Table S3).

In contrast, assigning individuals from the Atlantic back to their sampling location of origin was not accurate using all 79 SNPs (Figure S7A). Assignment accuracies were generally <20%, except for a few sites in which the mean accuracy ranged from 27% (Ber) to 58% (Vig) depending on the proportion of individuals used in the training data set. Accuracy was slightly higher when attempting to assign individuals from the Mediterranean back to their sampling location of origin (Figure S7B), although accuracy was generally still low and highly variable.

However, assigning individuals to one of three regions across the Atlantic genetic cline, informed by the DAPC and snapclust results, was more accurate (Figure 4b). When using all 103 individuals from the western North Sea (Eye‐Brd‐Cro, with Oosterschelde omitted due to its discrete differentiation), and 120 individuals randomly selected from Scandinavia (Hel‐Flo‐Sin‐Gul‐Kav‐Lys) and from the remaining Atlantic sites, all 79 SNPs assigned on average 60% of Scandinavian individuals and 63% of remaining Atlantic individuals to their correct region of origin (when the proportion of training individuals was 0.7). In addition, using only the top 10% of high *F*_st_ loci, assignment of Scandinavian individuals was improved across all training proportions (i.e. from 60% to 82% when the proportion of training individuals was 0.7), but assignment among the remaining Atlantic individuals was generally unchanged (Figure 4b). In contrast, individuals from the western North Sea consistently failed to assign to that region, instead tending to be split evenly between the Scandinavian group and the remaining Atlantic group. Assigning individuals to one of two regions in the Mediterranean was also more accurate (Figure 4c). When using all 32 individuals from the central Mediterranean (Sardinia and Lazio), and 50 individuals randomly selected from the Aegean Sea, all 79 SNPs assigned on average 88% of central Mediterranean individuals and 96% of Aegean Sea individuals to their correct region of origin (when the proportion of training individuals was 0.7).

## 4 DISCUSSION

This study comprises the first application of SNP markers isolated from RAD sequencing to investigate population genetic structure and assignment in the European lobster. Moreover, as opposed to sequencing multiple RAD libraries, this study isolated the most informative markers (in terms of maximizing *F*_st_ between our target groups) from one RAD screen and then genotyped those markers in all individuals at all sites sampled using a relatively inexpensive and high‐throughput SNP genotyping approach. This approach enabled a large number of individuals to be genotyped, increasing the likelihood of accurately representing allele frequencies at each site sampled, while still retaining a subset of informative SNPs to facilitate the exploration of basin‐wide patterns, and differentiation patterns at fine spatial scales in the geographical areas of highest population abundance (i.e. the north‐east Atlantic).

### 4.1 Basin‐wide genetic structure

This study revealed a pronounced phylogeographic break between the Atlantic and Mediterranean basins using 79 SNPs, a pattern detected by two previous studies of *H. gammarus* that used six allozymes (Jørstad et al., 2005) and RFLP analysis of a 3‐kb mtDNA segment (Triantafyllidis et al., 2005). However, compared to these two studies that similarly explored range‐wide genetic variation, the 79 SNPs from this study detected higher overall genetic differentiation (global *F*_st_ = 0.051 in this study, vs. 0.016 in Jørstad et al., 2005; global *G*_st_ = 0.106 in this study, vs. 0.078 in Triantafyllidis et al., 2005).

A partition between the Atlantic and the Mediterranean has also been found in previous studies for a diverse array of marine taxa, including other crustaceans (mtDNA, Reuschel, Cuesta, & Schubart, 2010; microsatellites, Palero, Abelló, Macpherson, Beaumont, & Pascual, 2011), molluscs (microsatellites, Pérez‐Losada, Guerra, Carvalho, Sanjuan, & Shaw, 2002), sponges (microsatellites, Riesgo et al., 2019), arrow worms (mtDNA and microsatellites, Peijnenburg, Fauvelot, Breeuwer, & Menken, 2006) and fish (mtDNA, Bargelloni et al., 2003). The majority of these studies ascribe this partition to restricted gene flow between the Atlantic and Mediterranean basins, most frequently due to IBD and/or an oceanographic barrier to connectivity. For example, Reuschel, Cuesta, & Schubart (2010) found a distinct phylogeographic break across the Atlantic–Mediterranean boundary in a littoral shrimp (*Palaemon elegans*), a finding the authors linked to reduced larval dispersal across the Almeria–Oran front, located in the western Mediterranean between Spain and Algeria. Although the Almeria–Oran front has been reported to impede gene flow in a number of marine species (Patarnello, Volckaert, & Castilho, 2007), the Strait of Gibraltar has also been implicated as a potential driver of genetic patterns (García‐Merchán et al., 2012). For instance, recurrent glaciations during the Pleistocene periodically reduced the width and depth of the Strait of Gibraltar (sea levels repeatedly decreased to ~120 m below present‐day levels; Rohling et al., 1998), which may have reduced connectivity between Atlantic and Mediterranean populations due to vicariance and habitat fragmentation (Charrier et al., 2006). In this study, very little admixture was detected between Atlantic and Mediterranean lobsters, although at putatively neutral SNPs some admixture was detected between the central Mediterranean and sites from the Atlantic, which appears to decrease as distance away from the central Mediterranean increases (Figures 2c and 3c). Overall, it is likely that the basin‐wide differentiation observed here has been shaped in part by both contemporary and historical barriers to gene flow and subsequent drift, possibly due to past/present oceanographic barriers and vicariance during the Pleistocene glaciations. Nevertheless, analysis with outlier SNPs also revealed strong basin‐wide differentiation (Figures 2b and 3b), which suggests that local adaptation to environmental conditions (e.g. sea temperature and salinity) may also contribute to the divergence of Atlantic–Mediterranean populations. However, due to a lack of samples from the western Mediterranean and from southern Iberia/northern Morocco in the Atlantic, it is difficult to ascertain the precise driver(s) of this basin‐wide differentiation in *H. gammarus*.

In addition to the strong differentiation observed between the Atlantic and Mediterranean basins, data from 79 SNPs identified differentiation (albeit slightly weaker) within the Mediterranean, separated into sites from the central Mediterranean (Sardinia and Lazio) and the Aegean Sea (all Greek sites). Furthermore, this pattern of differentiation was much stronger when analysed using only putatively neutral SNPs (Figures 2c and 3c). Although it cannot be discounted that this differentiation could be an artefact of ascertainment bias (since the original RAD screen contained no samples from the Aegean Sea), a similar pattern was found by Triantafyllidis et al. (2005), whereby *H. gammarus* samples from the Aegean Sea were differentiated from one site in the Adriatic Sea and from one site from the Columbretes Islands (in the western Mediterranean). The authors attributed this differentiation to the geographical isolation of Aegean Sea populations which could be caused by bathymetric and oceanographic conditions. Our study also found that populations from the Aegean Sea had in general the lowest values of observed heterozygosity compared with other sites sampled (Table 1), which may be indicative of past bottlenecks, possibly from natural mortality or as a result of historical overexploitation (Spanier et al., 2015). Overall, this suggests that the genetic differences observed between sites from Sardinia/Lazio and the Aegean Sea in this study are likely being driven by neutral drift, possibly via a combination of restricted connectivity (suggesting limited larval dispersal across this spatial scale) and historical contractions of effective population sizes.

### 4.2 North‐east Atlantic genetic structure

Across the north‐east Atlantic, a genetic cline is apparent, starting from Vigo in north‐west Spain to sites in Norway and Sweden (Figures 2 and 3), a pattern not detected by previous genetic studies of *H. gammarus*, and suggests that lobster populations across the north‐east Atlantic are not in complete panmixia. The most commonly proposed causes of clinal patterns in allele frequencies are as follows: (a) IBD caused by limited dispersal; (b) secondary contact and introgression between previously isolated and genetically divergent populations; and (c) selection across an environmental gradient (Dayan, 2018; Pérez‐Losada et al., 2002). Although much weaker using only neutral SNPs, a genetic cline was still evident in both the DAPC and the snapclust analyses (Figures 2c and 3c), and a significant association of genetic and geographical distances (Figure S4C) provides evidence for IBD in the north‐east Atlantic. Assuming IBD contributes to the formation of this cline, this would suggest that connectivity in *H. gammarus* follows a stepping‐stone model, as proposed by Ellis et al. (2017) based on the analysis of 14 microsatellite loci. However, the neutral clinal patterns observed in this study across the north‐east Atlantic could also be explained by expansion from refugia and secondary contact between previously isolated populations (Dayan, 2018). As range expansions and secondary contact are nonequilibrium processes, the clines produced can persist for many generations before they are eventually eroded by gene flow; thus, signatures of these processes may remain in the contemporary genetic structure of the marine organisms affected (Dayan, 2018). In the north‐east Atlantic, regional extirpation during the Last Glacial Maximum (LGM), followed by postglacial expansions, appears to be a common biogeographic history for many marine taxa (Jenkins, Castilho, & Stevens, 2018), although there is also evidence that some populations in ice‐free northern areas may have persisted in small periglacial refugia (Maggs et al., 2008). Putative southern refugia during the LGM (23–18 Ka) (Hewitt, 2004) have been proposed in south‐west Ireland (e.g. Assis, Serrao, Claro, Perrin, & Pearson, 2014; Hoarau, Coyer, Veldsink, Stam, & Olsen, 2007), the western English Channel (e.g. Assis et al., 2014), north‐west France (e.g. Coyer, Peters, Stam, & Olsen, 2003; Finnegan et al., 2013) and the Iberian Peninsula (e.g. Finnegan et al., 2013; Maggs et al., 2008), evidenced by the high levels of genetic diversity found in populations inhabiting these areas (Provan & Bennett, 2008). Given that samples from south‐west Ireland, south‐west England, western France and north‐west Spain yielded among the highest levels of observed heterozygosity in this study (Table 1), it is possible that these sites formed part of an area which served as a glacial refuge for *H. gammarus*, which preceded secondary contact of northward dispersers after the ice retreated.

The genetic cline, however, was even more distinctive when analysed with only the eight outlier SNPs (Figures 2b and 3b). Assuming that these SNP loci are indeed linked to or directly under the influence of selection, local adaptation across an environmental gradient cannot be ruled out as a causal factor of the cline. Indeed, evidence for local adaptation across both large spatial scales (i.e. ocean basins) and small spatial scales (i.e. within single estuaries) has been reported in numerous marine invertebrate species, of which sea temperature and salinity are key selective factors (Sanford & Kelly, 2011). As an example, a recent study reported a multispecies genetic cline in the north‐west Atlantic driven by sea temperature minima (Stanley et al., 2018); this study included a closely related species of *H. gammarus*, the American lobster (*Homarus americanus*), whose north‐west Atlantic range spans an extensive temperature gradient of −1°C to 26°C (Benestan, Quinn, et al., 2016). A similarly large thermal gradient exists across the range of *H. gammarus* populations sampled in this study, from the Aegean Sea (26°C maxima in summer) to the Skagerrak region (1°C minima in winter). Moreover, as with *H. americanus* (Quinn, Rochette, Ouellet, & Sainte‐Marie, 2013), temperature has been shown to be an important determinant of the development and behaviour of *H. gammarus* larvae during their pelagic dispersal phase (Schmalenbach & Franke, 2010). Although this provides some evidence that selection across an environmental gradient could explain the clinal patterns observed with outlier SNPs, additional analyses that incorporate environmental variables into the analysis are required to fully explore this hypothesis.

The results of this SNP study also indicated that lobsters from Oosterschelde are genetically differentiated from all other Atlantic sites analysed, which accords with previous studies (Jørstad et al., 2005; Triantafyllidis et al., 2005). Oosterschelde is a tidal estuarine system containing habitats such as intertidal flats, deep gullies, artificial rocky shores, and shallow water areas (Smaal, Kater, & Wijsman, 2009). During 1962–1963, harsh winters led to mass mortality of lobsters and other marine organisms in this area (Triantafyllidis et al., 2005), which would have drastically reduced effective population sizes. Indeed, Oosterschelde had one of the lowest measures of observed heterozygosity in this study (Table 1), and showed low haplotype diversity in Triantafyllidis et al. (2005), which supports a bottleneck scenario. In addition, construction of a storm surge barrier between the estuary and the North Sea was completed in 1986 to protect the area from flooding, although this is usually open to the North Sea so is not thought to be a permanent barrier to dispersal (Nienhuis & Smaal, 1994). Indeed, Smaal et al. (2009) reported that Pacific oysters, introduced into Oosterschelde as an exotic species from 1964, have expanded into the Wadden Sea, with northward larval dispersal from Oosterschelde among the most likely explanations for this colonization. Nevertheless, it is likely that past bottlenecks, and limited gene flow with adjacent North Sea sites, are responsible for the observed differentiation of Oosterschelde lobsters.

### 4.3 Assignment accuracy

The development and use of SNP panels composed of high‐ranking loci has proven to be extremely informative for assignment studies (e.g. Nielsen et al., 2012; Storer et al., 2012) and appears to offer particular promise for marine organisms showing weak overall genetic differentiation (Jorde, Synnes, Espeland, Sodeland, & Knutsen, 2018). In this study, the predictive model built using the baseline data composed of 79 SNPs was able to correctly assign 871 out of 874 (99.7%) “unknown” lobsters to their correct basin of origin. By comparison, across the north‐west Atlantic distribution of the American lobster, Benestan et al. (2015) were able to assign lobsters to north and south regions at ~94% accuracy, but only when using the top 3,000 most differentiated SNPs. The higher assignment accuracy in *H. gammarus* with substantially fewer SNP loci is likely reflective of the much higher differentiation observed between the north‐east Atlantic and the Mediterranean basins compared with the regional north–south differentiation in *H. americanus* across the north‐west Atlantic. This is not surprising considering the range‐wide *F*_st_ values generated for *H. gammarus* (0.051, this study) and *H. americanus* (0.002, Benestan et al., 2015), although the *F*_st_ calculation for *H. americanus* was based on 8,144 neutral SNPs for which no selection criteria were employed to maximize differentiation.

Assigning individuals of European lobster back to their sampling location of origin had low success in this study (generally <20%), which accords with the results for American lobster, whereby assignment success to population level achieved 25%–30% on average using 10,156 SNPs (Benestan, Gosselin, et al., 2016). In the present study, however, greater assignment success was achievable when individuals were assigned to intermediate regional scales. In the north‐east Atlantic, although accuracy was low when assigning individuals to the western North Sea, accuracy was much higher when assigning individuals to the remaining Atlantic sites (up to 63%) and the Scandinavian sites (up to 82%). This is reflective of the differentiation between the Scandinavian sites and the other Atlantic sites (excluding the western North Sea), which is evident from the pairwise differentiation statistics and the analyses of population structure in this study. Still, it cannot be ruled out that the overall lack of power for assigning individuals to region and location of origin may be a consequence of either true genetic homogeneity among sampling sites, or inadequate analytical power resulting from an insufficient number of markers and/or individuals per sample (the latter particularly for the assignment to sampling location, for which the maximum sample size was 40 individuals from Oosterschelde). This limitation was also outlined by Benestan et al. (2015), who suggested that substantially increasing the number of individuals per sampling location (at least 50 and ideally >100 individuals, Benestan, Gosselin, et al., 2016) could improve assignment success to these more precise spatial scales. In addition, assignment accuracy in this study may be improved by incorporating more SNP markers and by combining these extra data with software that attempts to account for clinal patterns of genetic variation (e.g. Drinan et al., 2018; Guillot, Jónsson, Hinge, Manchih, & Orlando, 2016).

### 4.4 Implications for management

Delineating conservation units is a fundamental requirement for fisheries and conservation managers, so that they recognize the boundaries of the populations they are trying to preserve (Funk et al., 2012; Palsbøll et al., 2007). Evolutionary significant units (ESUs) typically consider all the genetic variation among a sample of populations, while management units (MUs) and adaptive units (AUs) usually consider only neutral and adaptive genetic variation, respectively (Barbosa et al., 2018; Funk et al., 2012). The results from this SNP study indicate that two overarching ESUs exist across the range of the European lobster, partitioned between populations from the north‐east Atlantic and populations from the Mediterranean. Overall, however, it appears there are (based on the sampling undertaken in this study) at least two distinct ESUs in the Mediterranean, divided into the central Mediterranean and the Aegean Sea, and at least three in the north‐east Atlantic; this is represented by two units that show a longitudinal clinal pattern, whereby genetic distinctiveness is highest between Vigo (north‐west Spain) and Scandinavia, and one from Oosterschelde.

The results from this study using putatively neutral SNPs, combined with the results from previous studies (Ellis et al., 2017; Jørstad et al., 2005; Triantafyllidis et al., 2005), suggest that gene flow in *H. gammarus* across the north‐east Atlantic likely follows a stepping‐stone model of connectivity. If true, this implies that site‐specific recruitment may not always come from local sources, but potentially from adjacent local or regional sources. Thus, a localized depletion of abundance may in fact reduce recruitment in adjacent stocks and potentially cause a more far‐reaching depletion across surrounding fisheries. Recent research has found that temporary closures or prohibiting fishing in marine protected areas (MPAs) offers some respite to lobster populations (Moland et al., 2013; Roach, Cohen, Forster, Revill, & Johnson, 2018; Sørdalen et al., 2018), highlighting their viability as a management option to prevent overexploitation of lobster fisheries. However, although safeguarding lobster stocks via temporary closures or MPAs may increase size and density of lobsters in reserves in the short term, the value of larval spillover from these reserves to surrounding areas is potentially just as important but requires the design and implementation of longer‐term management strategies to be effective.

For lobster hatcheries, knowledge of stock structure is crucial to ensure that juveniles, which are usually reared from the egg clutches of wild‐mated females (Ellis et al., 2015), are genetically compatible with the target population being stocked (Ward, 2006). Overall, the genetic profiles observed in this study suggest that stock enhancement and restocking should ideally be implemented with juveniles whose parents originate from the same geographical region. Furthermore, the use of broodstock originating from the north‐east Atlantic to restock populations in the Mediterranean, or vice versa, is highly discouraged because of the potential to introduce maladapted traits into the target population that could also proliferate to neighbouring populations (Araki, Cooper, & Blouin, 2007). The futility of stocking with exogenous broodstock has been amply demonstrated in Atlantic salmon (Finnegan & Stevens, 2008; Griffiths et al., 2011), and lobster hatcheries should mitigate the propensity for unwanted side effects if release programs are to achieve conservation ambitions (Ellis et al., 2015).

Individual assignment using genetic techniques has been shown to be a potentially useful tool for determining the origin of fished individuals and for tackling illegal fishing (Bernatchez et al., 2017; Martinsohn & Ogden, 2009; Nielsen et al., 2012). However, the power of the markers employed is highly sensitive to the degree of genetic differentiation between sites (Christie, Meirmans, Gaggiotti, Toonen, & White, 2017). This study demonstrated that a panel of 79 SNPs has adequate power to assign lobsters accurately to either the Atlantic or the Mediterranean basin. This may have useful applications for management authorities, such as estimating the proportions of native versus imported European lobster consumed as seafood in the Mediterranean, or to ensure that any attempts to restock depleted Mediterranean areas utilize local broodstock. Moreover, managers could test for the presence of Atlantic‐origin lobsters in the Mediterranean via escaped or released animals. At present, though, it is not possible to accurately assign lobsters back to their precise location of origin using the SNP panel employed in this study. Nevertheless, it may be possible to assign lobsters with some confidence to geographical regions (e.g. Scandinavia) which could have useful applications for similar reasons.

### 4.5 Limitations and conclusions

This study conducted analyses of population structure on all SNPs, but also independently on putatively neutral and outlier SNPs to facilitate the inference of neutral versus adaptive processes in driving the genetic patterns observed. However, because the outlier selection tests were carried out on the original RAD sequencing data set (composed of 55 individuals), this may have reduced the power to detect genuine outliers because many sites included in this SNP study were not included in the original RAD analysis. All outliers detected originated from the SNP loci selected for the panel to maximize differentiation between the Atlantic, Skagerrak and the Mediterranean (Figure S1; Jenkins et al., 2018), of which five were removed in the current study because of LD (Table S2; Figure S2). Of these outlier SNPs, one (65064) had low identity matches on BLASTx, which is likely a product of the general lack of well‐annotated genomic resources for marine decapods. Interestingly, outlier SNPs were informative for both the north‐east Atlantic genetic cline and for the differentiation between the Atlantic and Mediterranean basins (Figures 2b and 3b). This may indicate that some of these outlier loci, particularly SNPs 42395 and 53935 (Figure 5), and SNP 65064 and the loci in LD with SNP 65064 (Figure S2), have undergone parallel genetic divergence (i.e. convergence of allele frequency patterns; Bierne, Gagnaire, & David, 2013). A similar pattern was found in six outlier SNPs in long‐snouted seahorses (Riquet et al., 2019), whereby genetic parallelism between a Mediterranean lagoon ecotype and a north Atlantic lineage was detected at a large genomic island. Mapping the location of the outlier SNPs and the SNPs in LD in this study would allow us to discern whether some (or all) of these SNPs are also located in a genomic island of differentiation, but at present a reference genome assembly for *H. gammarus* or *H. americanus* is not available.

In conclusion, using 79 SNPs selected for their ability to maximize genetic differentiation at a range of both broad and fine scales, this study found that basin‐wide patterns of population structure (i.e. differentiation between the Atlantic and Mediterranean basins) generally accord with previous genetic studies of *H. gammarus*, but, uniquely, the additional resolution provided by this study revealed a genetic cline across the north‐east Atlantic. Analyses of neutral SNPs suggested that this cline could have been produced in part by IBD or secondary contact, or both, as there is evidence that restrictions in contemporary gene flow can maintain neutral nonequilibrium clines formed by postglacial expansions and secondary contact (Dayan, 2018). However, analysis with outlier SNPs suggests local adaptation across an environmental gradient (e.g. temperature) cannot be ruled out as a causal factor of the genetic cline. In contrast to previous studies that employed traditional genetic markers (e.g. microsatellites), this SNP‐based study detected far greater levels of genetic differentiation. As a result of the higher differentiation detected, the predictive model assembled was able to assign 99.7% of “unknown” lobsters (lobsters whose origin was known but were omitted from the baseline data set) to their correct basin of origin (Atlantic or Mediterranean), although the accuracy of this method decreased when attempting to assign to region of origin and again when assigning to sampling location. Importantly, from an applied perspective, as these genetic patterns were uncovered using a SNP panel designed for high‐throughput performance, genotyping additional lobster DNA samples can be done rapidly (96 samples in ~6 hr using a Fluidigm EP1 system) and relatively inexpensively. This has important benefits for future analyses of *H. gammarus* genetic structure, as new individuals and sampling sites can be added to form larger spatial and temporal SNP data sets without the need for further cross‐calibration, which has previously proved highly problematic in studies of other species using microsatellite markers (Ellis et al., 2011). In addition, we envisage that this panel of SNPs will be useful as a traceability tool for seafood and aquaculture industries for establishing the mesoscale origins of European lobsters.

## CONFLICT OF INTEREST

None declared.

## Data Availability

Data for this study are available at the Dryad Digital Repository: https://doi.org/10.5061/dryad.2v1kr38.
