# Supplementary material for: Single nucleotide polymorphisms reveal a genetic cline across the north‐east Atlantic and enable powerful population assignment in the European lobster
Source: Evol Appl. 2019 Aug 7;12(10):1881–99. doi: 10.1111/eva.12849 (PMC6824076; doi:10.1111/eva.12849)
Supplement: Supplementary file 1 [file EVA-12-1881-s001.pdf]

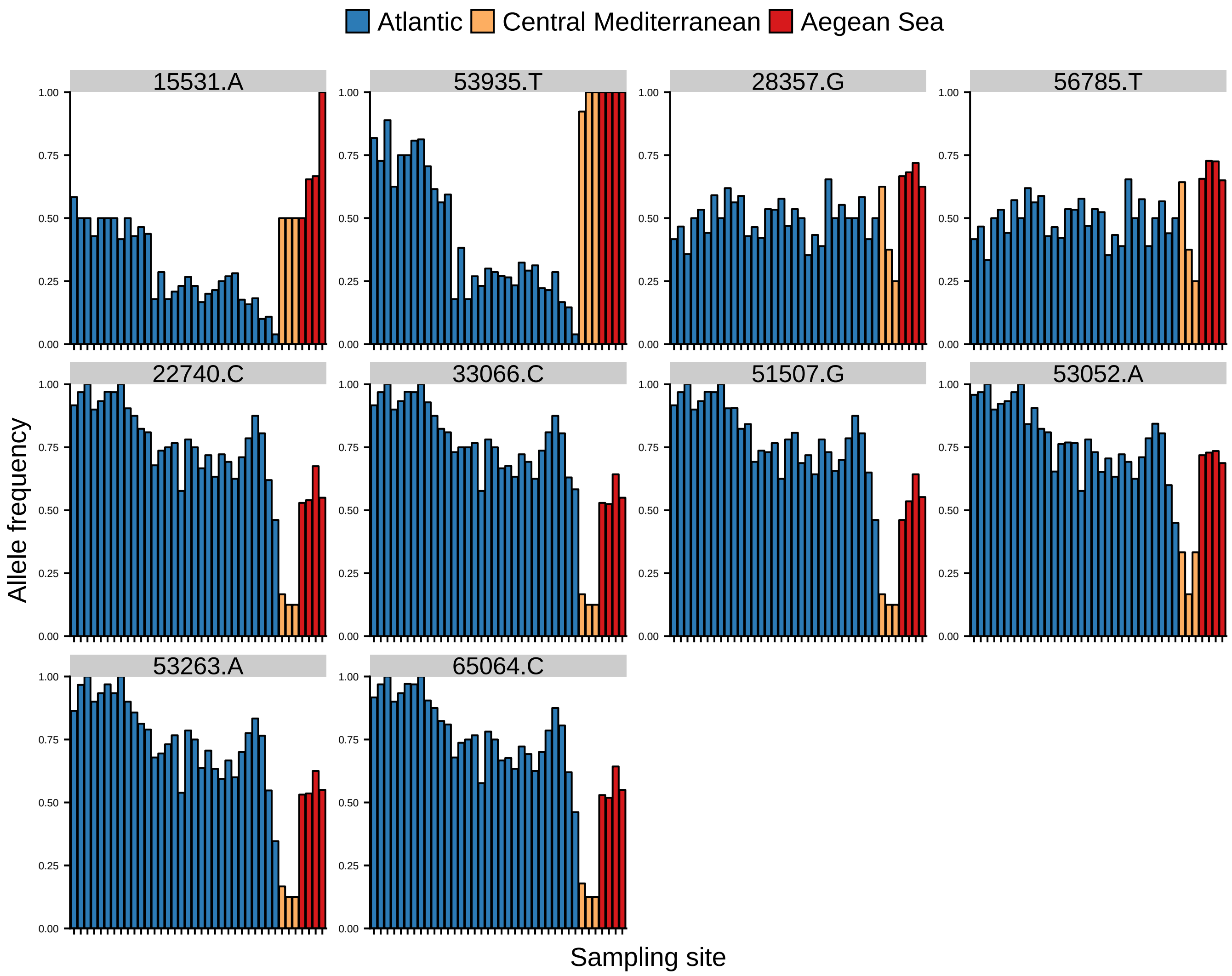

Figure S2 Population allele frequency of one allele for each SNP in which linkage disequilibrium (LD) was detected. Significant LD was detected between SNPs 15531 and 53935, between SNPs 28357 and 56785, and between SNPs 22740, 33066, 51507, 53052, 53263 and 65064. For each SNP, the sampling sites (x-axis) are arranged in the following order: Tro, Ber, Flo, Gul, Kav, Lys, Sin, Hel, Oos, Cro, Brd, Eye, She, Ork, Heb, Sul, Cor, Hoo, Iom, Ios, Jer, Kil, Loo, Lyn, Mul, Pad, Pem, Sbs, Ven, Idr, Vig, Sar, Laz, Tar, Ale, Sky, The, Tor. Colours denote whether the sampling site originates from the Atlantic, the central Mediterranean or the Aegean Sea.
