# Supplementary material for: Single nucleotide polymorphisms reveal a genetic cline across the north‐east Atlantic and enable powerful population assignment in the European lobster
Source: Evol Appl. 2019 Aug 7;12(10):1881–99. doi: 10.1111/eva.12849 (PMC6824076; doi:10.1111/eva.12849)
Supplement: Supplementary file 2 [file EVA-12-1881-s002.pdf]

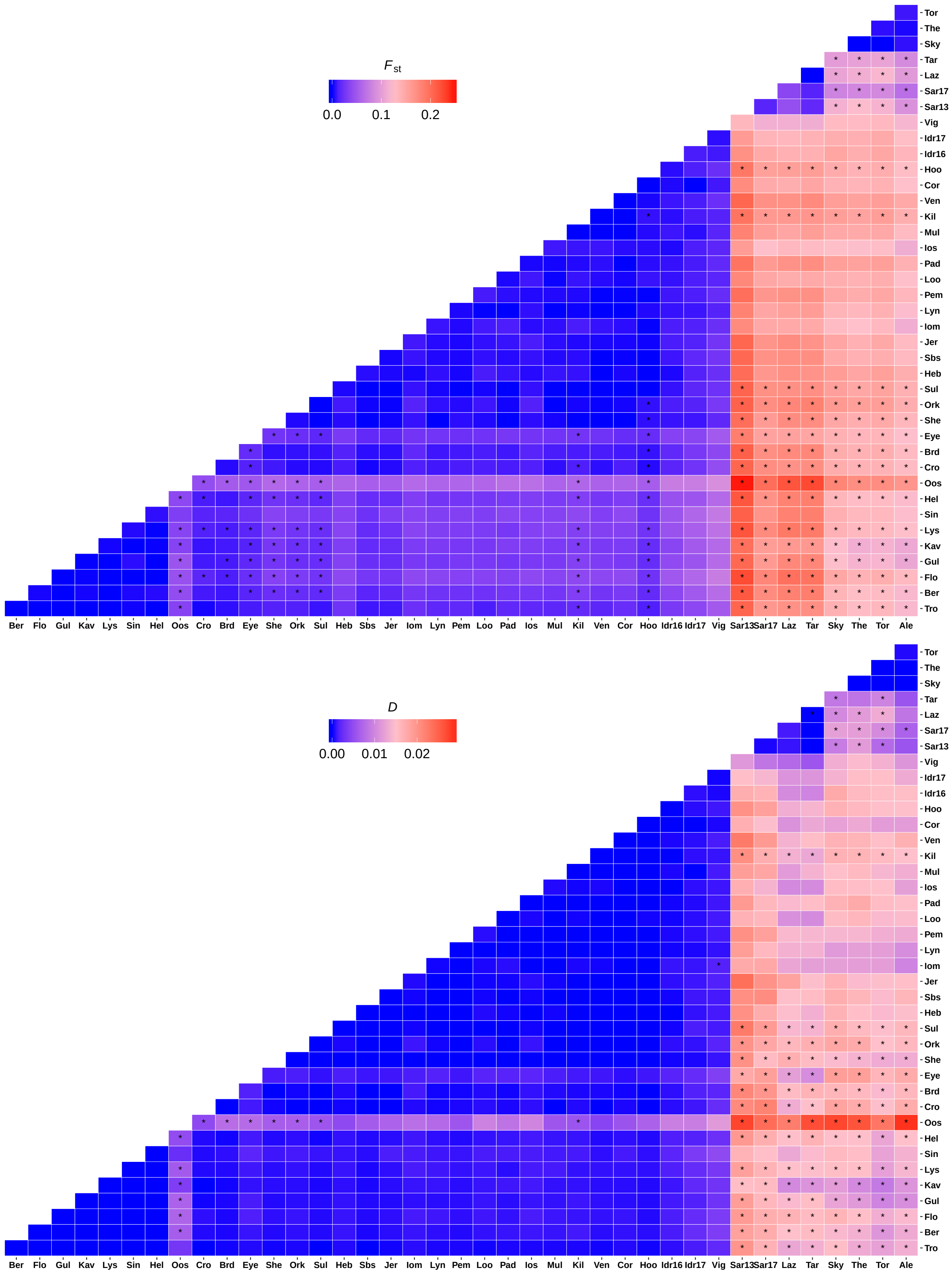

Figure S3 European lobster heatmaps of F_{st} (top) and Jost's D (bottom). Asterisks represent pairwise comparisons that were significantly different from zero.
