# Supplementary material for: Single nucleotide polymorphisms reveal a genetic cline across the north‐east Atlantic and enable powerful population assignment in the European lobster
Source: Evol Appl. 2019 Aug 7;12(10):1881–99. doi: 10.1111/eva.12849 (PMC6824076; doi:10.1111/eva.12849)
Supplement: Supplementary file 3 [file EVA-12-1881-s003.pdf]

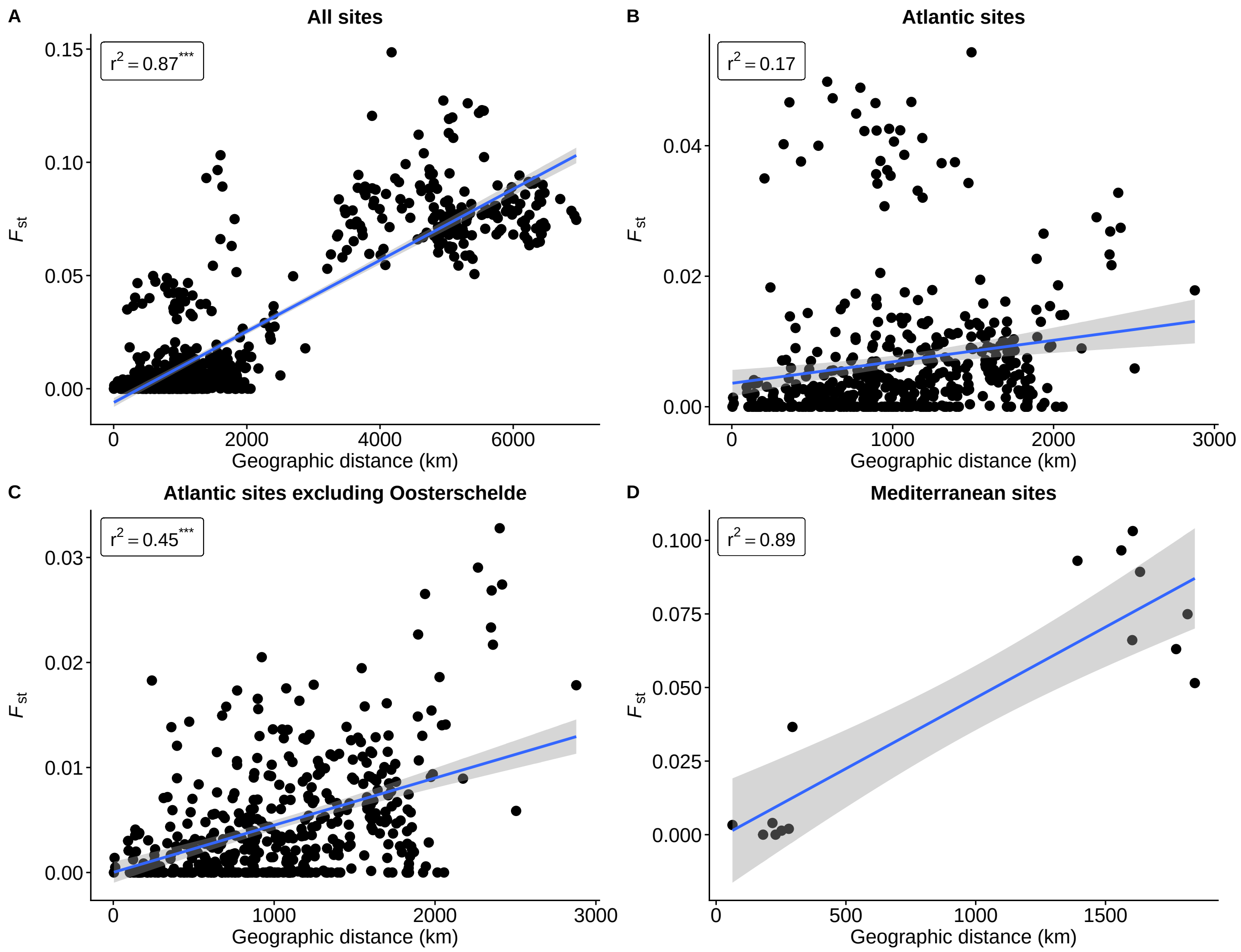

Figure S4 Pairwise comparisons of geographic distances (km) and F_{st} between European lobster sampling sites. Mantel tests were conducted using all sites (A), using only Atlantic sites (B), using only Atlantic sites excluding Oosterschelde, and using only Mediterranean sites (D). For each plot, a linear regression line (in blue) is fitted with 95 % confidence intervals (in grey). Asterisks denote significance levels: *<0.05, **<0.01, ***<0.001.
