# Supplementary material for: Single nucleotide polymorphisms reveal a genetic cline across the north‐east Atlantic and enable powerful population assignment in the European lobster
Source: Evol Appl. 2019 Aug 7;12(10):1881–99. doi: 10.1111/eva.12849 (PMC6824076; doi:10.1111/eva.12849)
Supplement: Supplementary file 4 [file EVA-12-1881-s004.pdf]

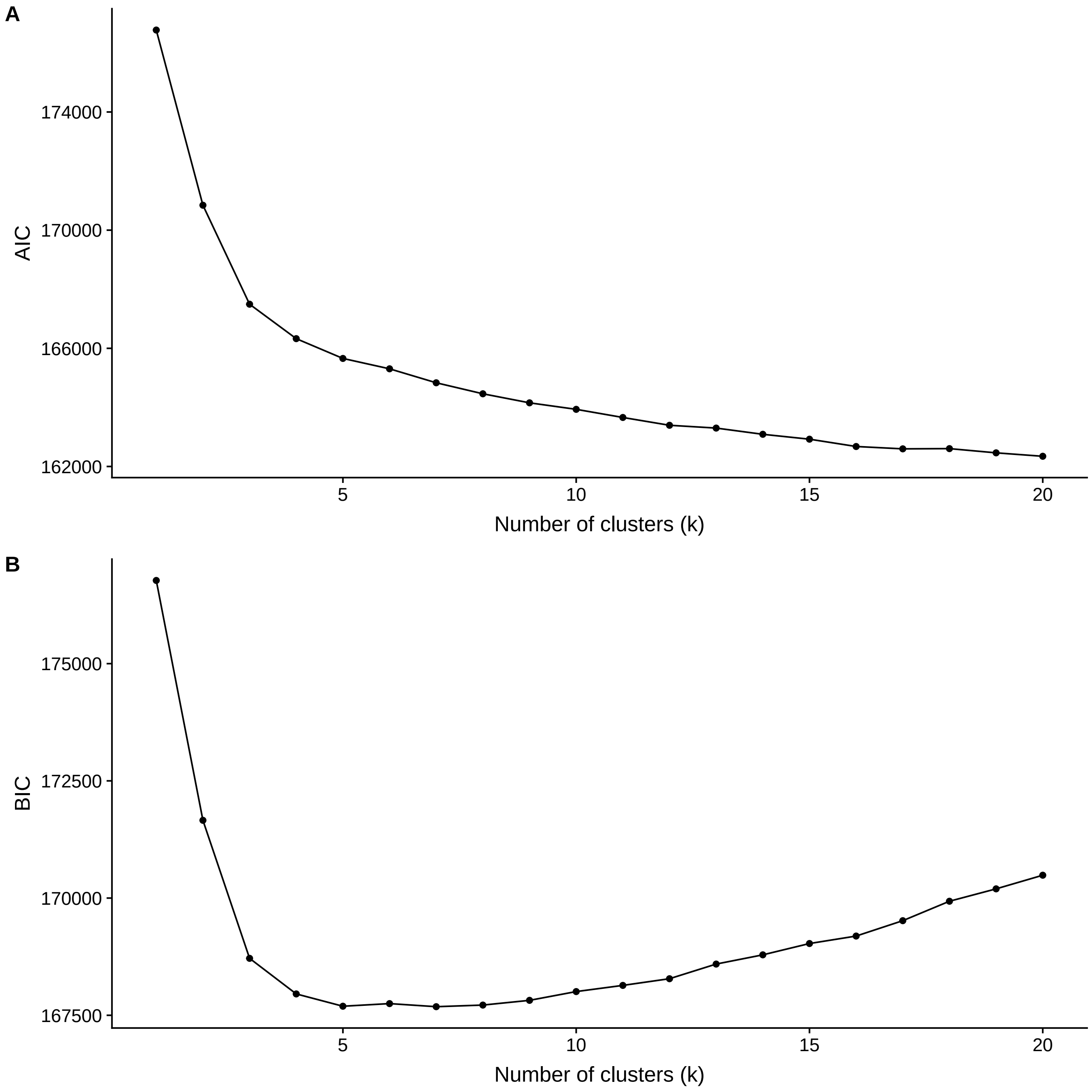

Figure S5 Snapclust goodness-of-fit statistics: (A) Akaike information criterion (AIC) using all 79 SNPs, (B) Bayesian information criterion (BIC) using all 79 SNPs.

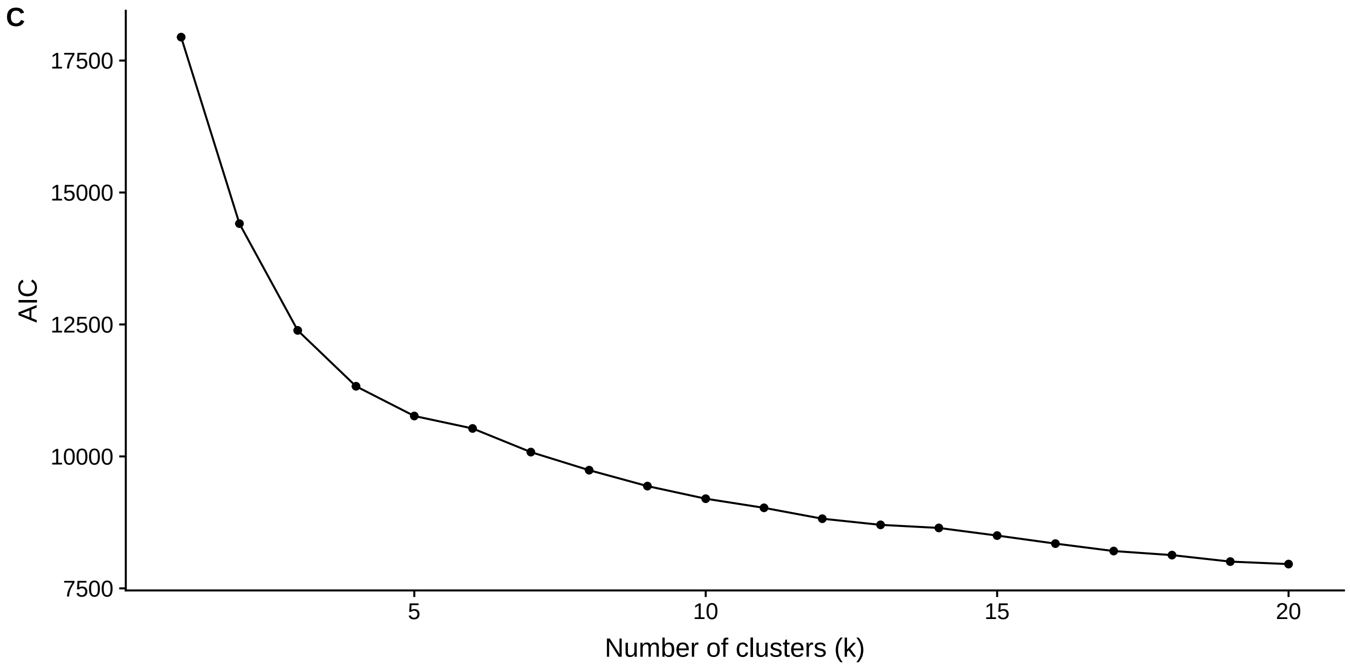

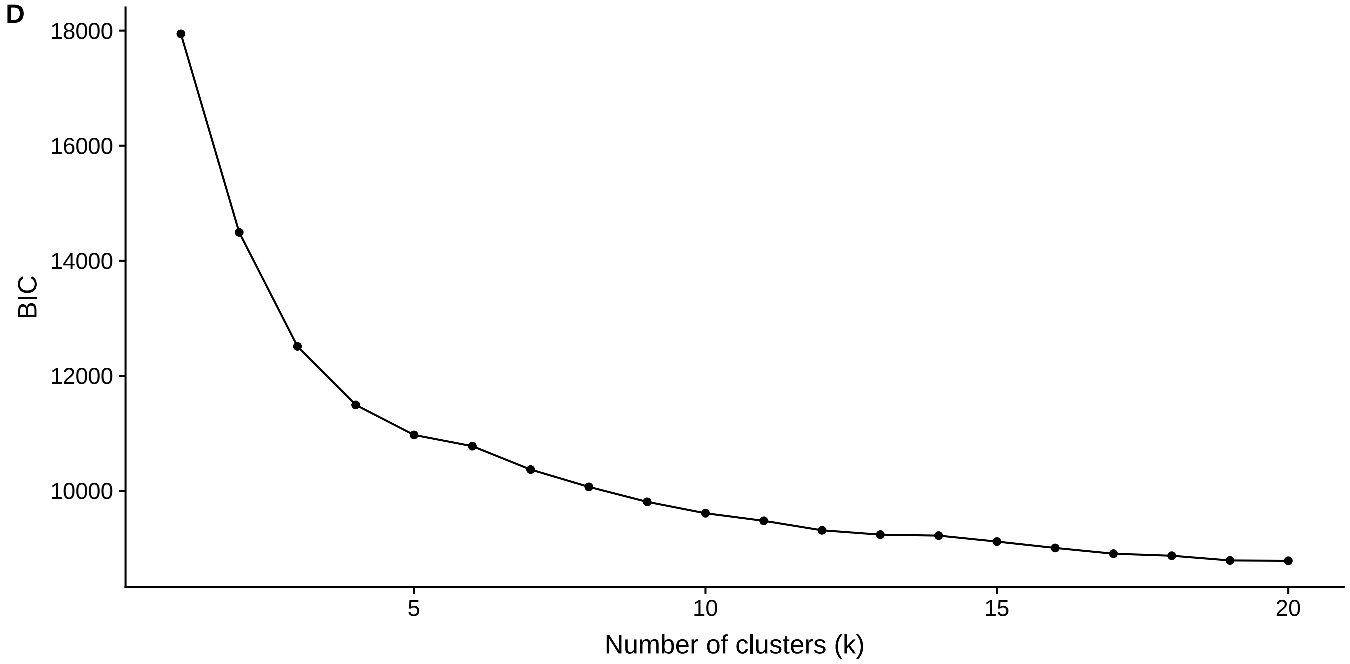

Figure S5 Snapclust goodness-of-fit statistics: (C) Akaike information criterion (AIC) using eight outlier SNPs, (D) Bayesian information criterion (BIC) using eight outlier SNPs.

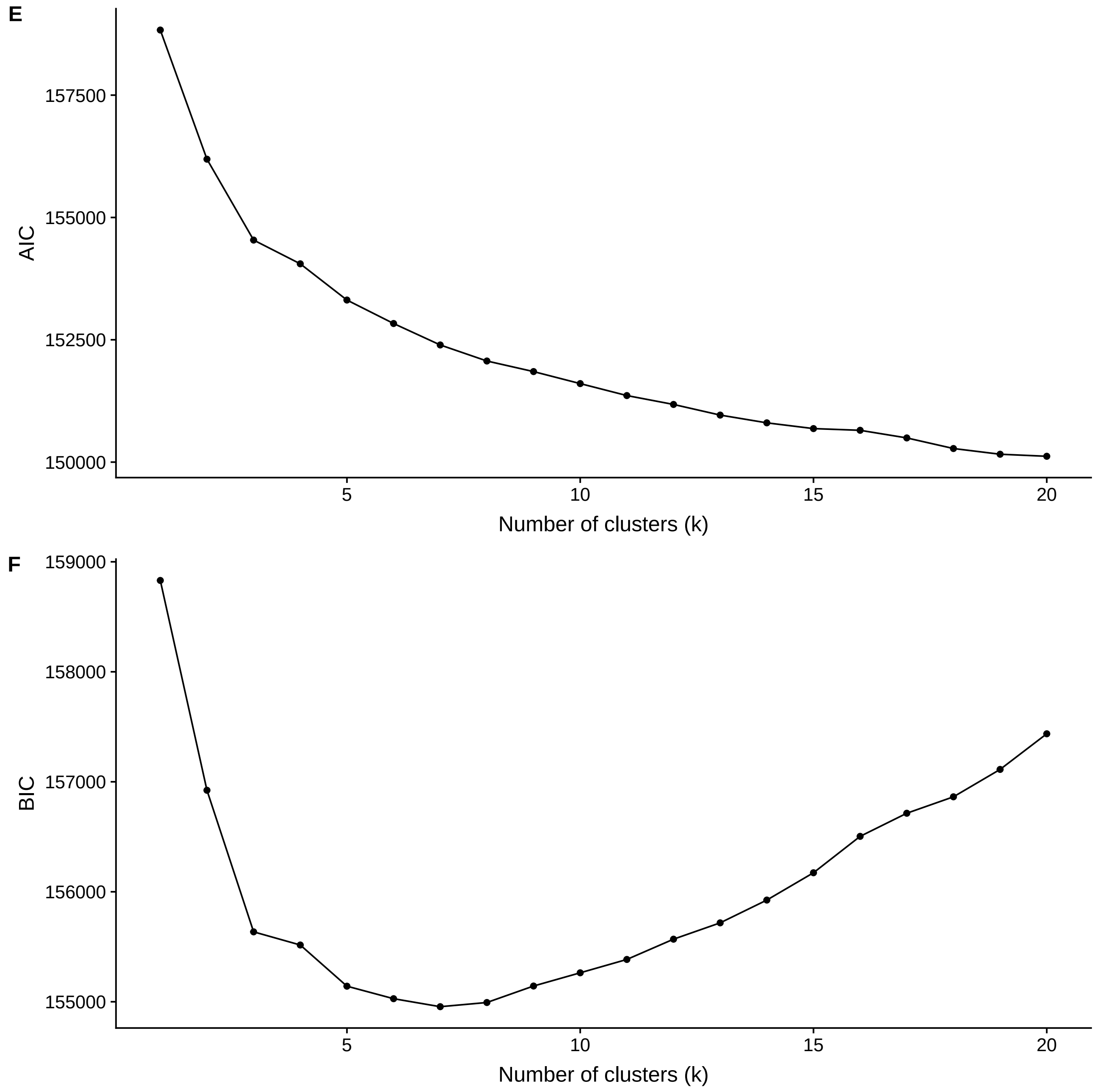

Figure S5 Snapclust goodness-of-fit statistics: (E) Akaike information criterion (AIC) using 71 neutral SNPs, (F) Bayesian information criterion (BIC) using 71 neutral SNPs.
