# Supplementary material for: Single nucleotide polymorphisms reveal a genetic cline across the north‐east Atlantic and enable powerful population assignment in the European lobster
Source: Evol Appl. 2019 Aug 7;12(10):1881–99. doi: 10.1111/eva.12849 (PMC6824076; doi:10.1111/eva.12849)
Supplement: Supplementary file 5 [file EVA-12-1881-s005.pdf]

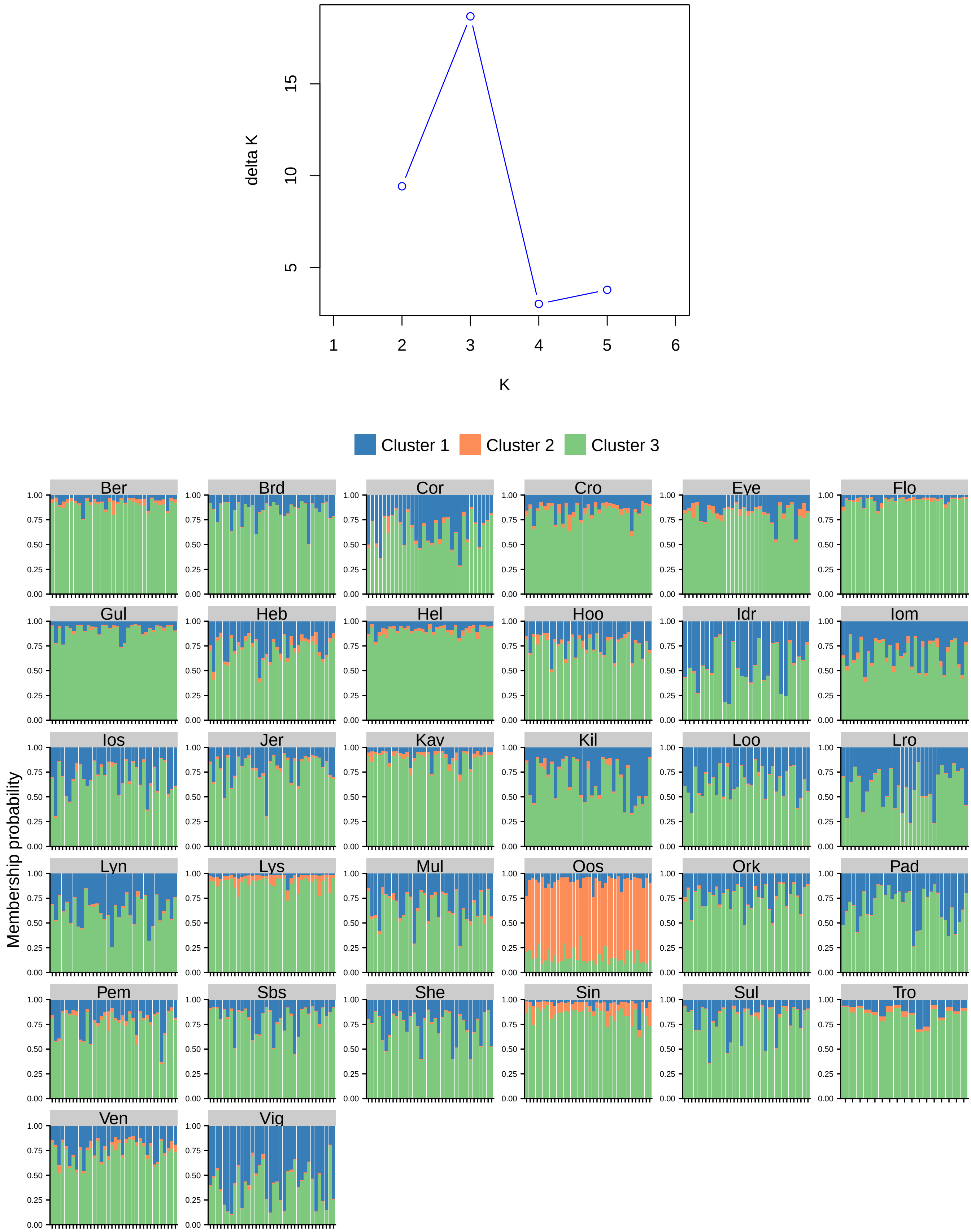

Figure S6 Atlantic hierarchical STRUCTURE analysis using 71 neutral SNPs. The delta *K* statistic suggested *K*=3 was most informative *K* for this dataset (top). STRUCTURE results are shown for each sampling site (bottom). Each bar represents an individual and colours denote membership proportions to each cluster.
