# Supplementary material for: Single nucleotide polymorphisms reveal a genetic cline across the north‐east Atlantic and enable powerful population assignment in the European lobster
Source: Evol Appl. 2019 Aug 7;12(10):1881–99. doi: 10.1111/eva.12849 (PMC6824076; doi:10.1111/eva.12849)
Supplement: Supplementary file 6 [file EVA-12-1881-s006.pdf]

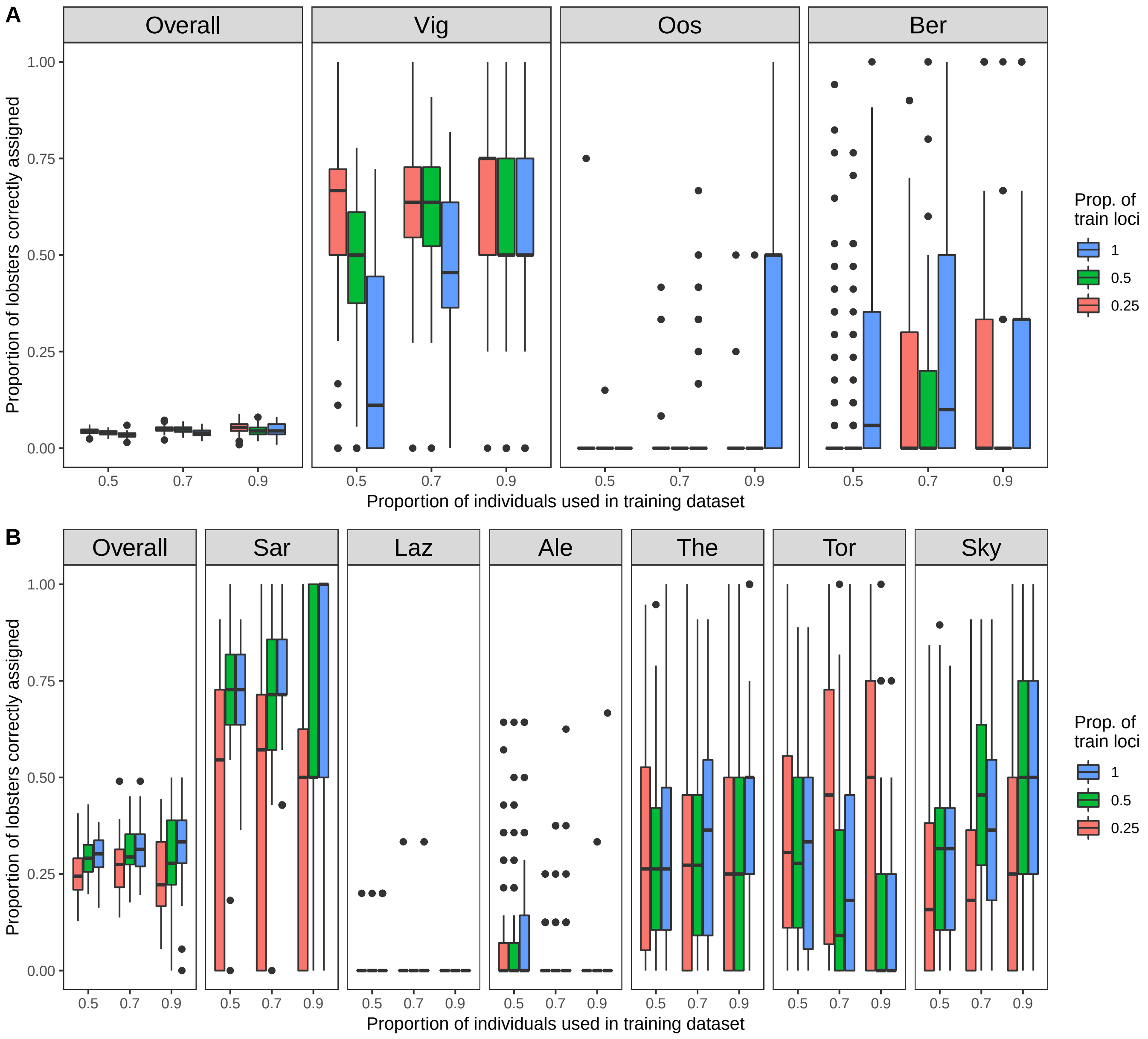

Figure S7 Assignment accuracies estimated via Monte-Carlo cross-validation, with three levels of training (baseline) individuals (50 %, 70 % and 90 % of individuals from each group) crossed by up to three levels of training loci (25 %, 50 % and all loci) by 100 resampling events: (A) Atlantic location of origin analysis; (B) Mediterranean location of origin analysis.
