# Supplementary material for: Single nucleotide polymorphisms reveal a genetic cline across the north‐east Atlantic and enable powerful population assignment in the European lobster
Source: Evol Appl. 2019 Aug 7;12(10):1881–99. doi: 10.1111/eva.12849 (PMC6824076; doi:10.1111/eva.12849)
Supplement: Supplementary file 7 [file EVA-12-1881-s007.docx]

**Table S2: Outlier selection tests on the raw RADseq data from Jenkins et al. (2018)**

Outlier selection tests were carried out on the full RADseq dataset (7,022 SNPs) and the Atlantic RADseq dataset (4,377 SNPs) from Jenkins et al. (2018). This document contains a table showing which of the 96 SNPs selected for the SNP panel in Jenkins et al. (2018) were identified as an outlier by Bayescan, OutFLANK and PCAdapt.

**Key:**

|  |
| --- |
|  |

**SNP identified as an outlier.**

**SNP identified as an outlier in two or more selection tests.**

***SNP matched one or more translated proteins on BLASTx.**

**^EP1^Did not work consistently on the Fluidigm EP1 system.**

**^MISS^Contained missing data.**

**^HWE^Removed due to deviation from Hardy-Weinberg equilibrium.**

**^Hobs^Removed due to unexpectedly high proportion of heterozygosity.**

**^LD^Removed due to linkage disequilibrium.**

| **SNP locus ID** | **Dataset origin** | **Bayescan** | **OutFLANK** | **PCAdapt** |
| --- | --- | --- | --- | --- |
| 6157 | Full |  |  |  |
| 11291 | Full |  |  |  |
| 15128 | Full |  |  |  |
| 15531**^LD^** | Full |  |  |  |
| 15581 | Full |  |  |  |
| 22740**^LD^** | Full |  |  |  |
| 29889 | Full |  |  |  |
| 31462 | Full |  |  |  |
| 32358 | Full |  |  |  |
| 33066**^LD^** | Full |  |  |  |
| 42395 | Full |  |  |  |
| 44670 | Full |  |  |  |
| 51507**^LD^** | Full |  |  |  |
| 53052**^LD^** | Full |  |  |  |
| 53263**^LD^*** | Full |  |  |  |
| 53314 | Full |  |  |  |
| 53935 | Full |  |  |  |
| 58053 | Full |  |  |  |
| 59586 | Full |  |  |  |
| 65064***** | Full |  |  |  |
| 65576 | Full |  |  |  |
| 3441 | Atlantic |  |  |  |
| 4173 | Atlantic |  |  |  |
| 7502 | Atlantic |  |  |  |
| 7892 | Atlantic |  |  |  |
| 8953**^HWE^** | Atlantic |  |  |  |
| 9441 | Atlantic |  |  |  |
| 11071 | Atlantic |  |  |  |
| 11183 | Atlantic |  |  |  |
| 12971 | Atlantic |  |  |  |
| 14047 | Atlantic |  |  |  |
| 14742 | Atlantic |  |  |  |
| 15109 | Atlantic |  |  |  |
| 15435 | Atlantic |  |  |  |
| 18512 | Atlantic |  |  |  |
| 18652 | Atlantic |  |  |  |
| 19266 | Atlantic |  |  |  |
| 19460 | Atlantic |  |  |  |
| 20354 | Atlantic |  |  |  |
| 21197**^HWE^** | Atlantic |  |  |  |
| 21880**^Hobs^** | Atlantic |  |  |  |
| 22323**^Hobs^** | Atlantic |  |  |  |
| 22365**^MISS^** | Atlantic |  |  |  |
| 23146 | Atlantic |  |  |  |
| 23447 | Atlantic |  |  |  |
| 23481 | Atlantic |  |  |  |
| 23677 | Atlantic |  |  |  |
| 23787 | Atlantic |  |  |  |
| 24020 | Atlantic |  |  |  |
| 25229 | Atlantic |  |  |  |
| 25580**^EP1^** | Atlantic |  |  |  |
| 25608 | Atlantic |  |  |  |
| 27329 | Atlantic |  |  |  |
| 28357**^LD^** | Atlantic |  |  |  |
| 29410 | Atlantic |  |  |  |
| 29801 | Atlantic |  |  |  |
| 30339 | Atlantic |  |  |  |
| 31618 | Atlantic |  |  |  |
| 31967 | Atlantic |  |  |  |
| 31979 | Atlantic |  |  |  |
| 32362**^EP1^** | Atlantic |  |  |  |
| 32435 | Atlantic |  |  |  |
| 33784 | Atlantic |  |  |  |
| 34443 | Atlantic |  |  |  |
| 34818 | Atlantic |  |  |  |
| 35584 | Atlantic |  |  |  |
| 36910 | Atlantic |  |  |  |
| 39107 | Atlantic |  |  |  |
| 39876 | Atlantic |  |  |  |
| 41521**^EP1^** | Atlantic |  |  |  |
| 42529 | Atlantic |  |  |  |
| 42821 | Atlantic |  |  |  |
| 45154 | Atlantic |  |  |  |
| 45217 | Atlantic |  |  |  |
| 51159 | Atlantic |  |  |  |
| 53720 | Atlantic |  |  |  |
| 53889**^EP1^** | Atlantic |  |  |  |
| 54240 | Atlantic |  |  |  |
| 54762 | Atlantic |  |  |  |
| 55111 | Atlantic |  |  |  |
| 55142 | Atlantic |  |  |  |
| 55564 | Atlantic |  |  |  |
| 56423 | Atlantic |  |  |  |
| 56785 | Atlantic |  |  |  |
| 57131 | Atlantic |  |  |  |
| 57989 | Atlantic |  |  |  |
| 59503 | Atlantic |  |  |  |
| 59967 | Atlantic |  |  |  |
| 60546 | Atlantic |  |  |  |
| 63140 | Atlantic |  |  |  |
| 63267 | Atlantic |  |  |  |
| 63581 | Atlantic |  |  |  |
| 63605 | Atlantic |  |  |  |
| 63771 | Atlantic |  |  |  |
| 63798 | Atlantic |  |  |  |
| 65376**^EP1^** | Atlantic |  |  |  |

**References**

Jenkins TL, Ellis CD, Stevens JR (2018) SNP discovery in European lobster (*Homarus gammarus*) using RAD sequencing. *Conservation Genetics Resources*, https://doi.org/10.1007/s12686-018-1001-8.
