# Supplementary material for: Single nucleotide polymorphisms reveal a genetic cline across the north‐east Atlantic and enable powerful population assignment in the European lobster
Source: Evol Appl. 2019 Aug 7;12(10):1881–99. doi: 10.1111/eva.12849 (PMC6824076; doi:10.1111/eva.12849)
Supplement: Supplementary file 9 [file EVA-12-1881-s009.pdf]

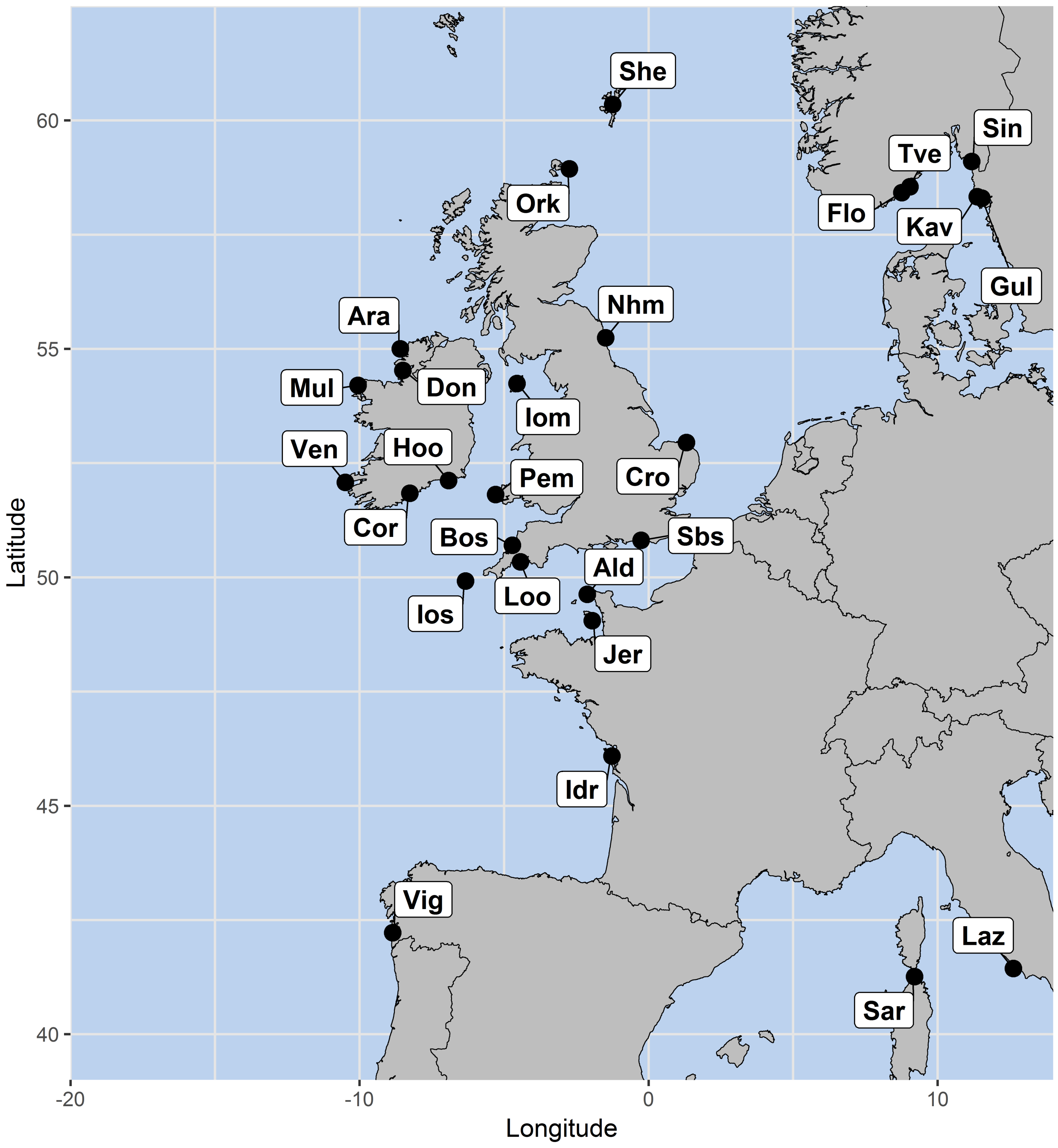

Figure S1A Sampling sites included in the RAD sequencing of European lobster.

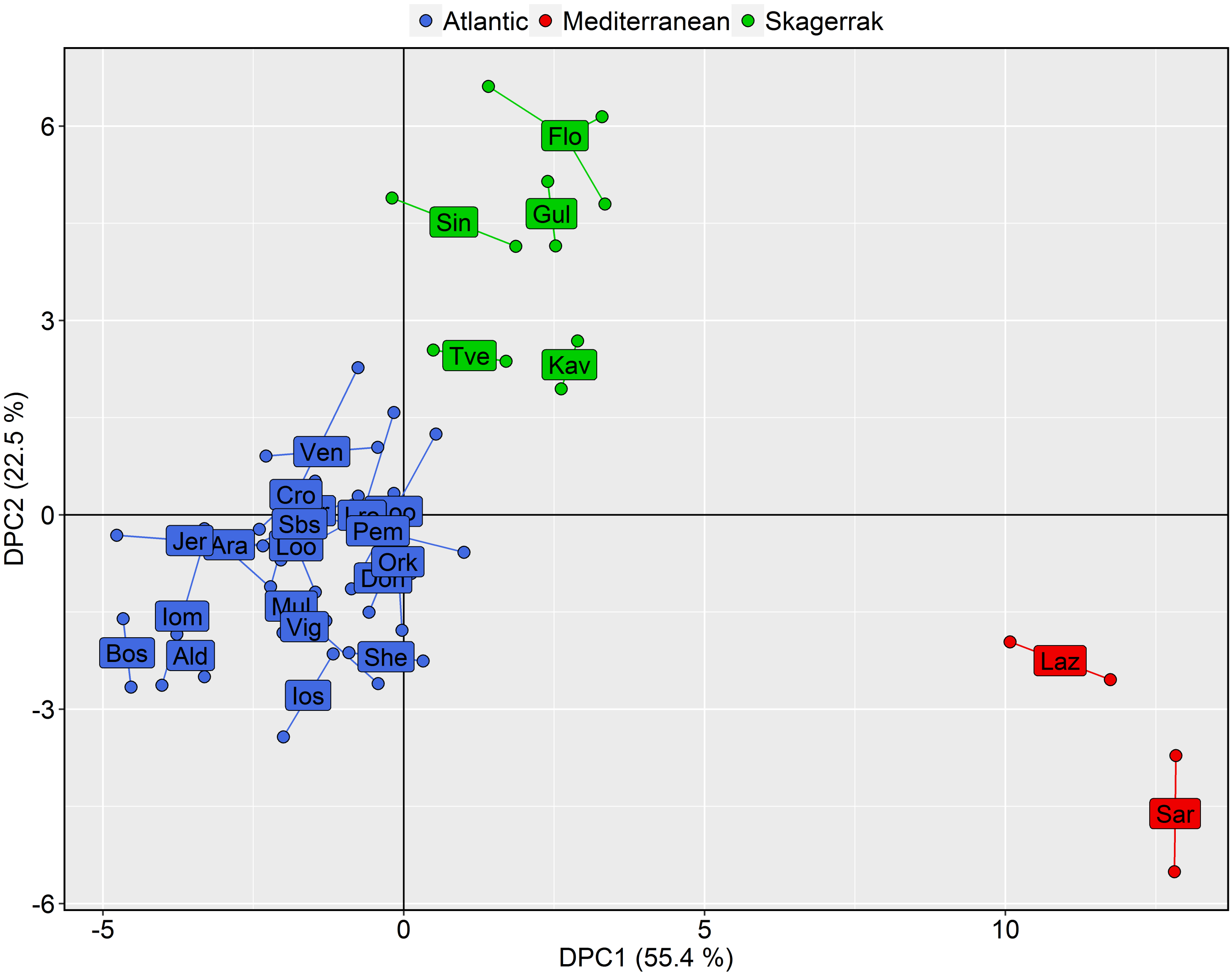

Figure S1B Discriminant analysis of principal components using 55 individuals and 7,022 SNPs.

Table S1 Stacks population map composed of nine putative populations and 40 individuals.

**Population**	**Individuals**	***N***
English Channel	Ald3, Ald4, Jer2, Jer3, Sbs2, Sbs3	6
West Ireland	Ara1, Ara2, Don2, Don3, Ven1, Ven3, Mul3, Mul4	8
Southwest England	Bos55, Bos67, Ios5, Ios6, Loo13_31, Loo16_18	6
Southeast Ireland	Cor1, Cor2, Hoo1, Hoo2	4
North Sea	Cro4, Cro5, Nhm8	3
Irish Sea	Iom1, Iom4, Pem12, Pem14	4
Orkney & Shetland	Ork15_6, Ork16_1, She14_3, She14_4	4
France	Lro_4, Idr16_11, Idr16_13	3
Spain	Vig13_1, Vig13_3	2

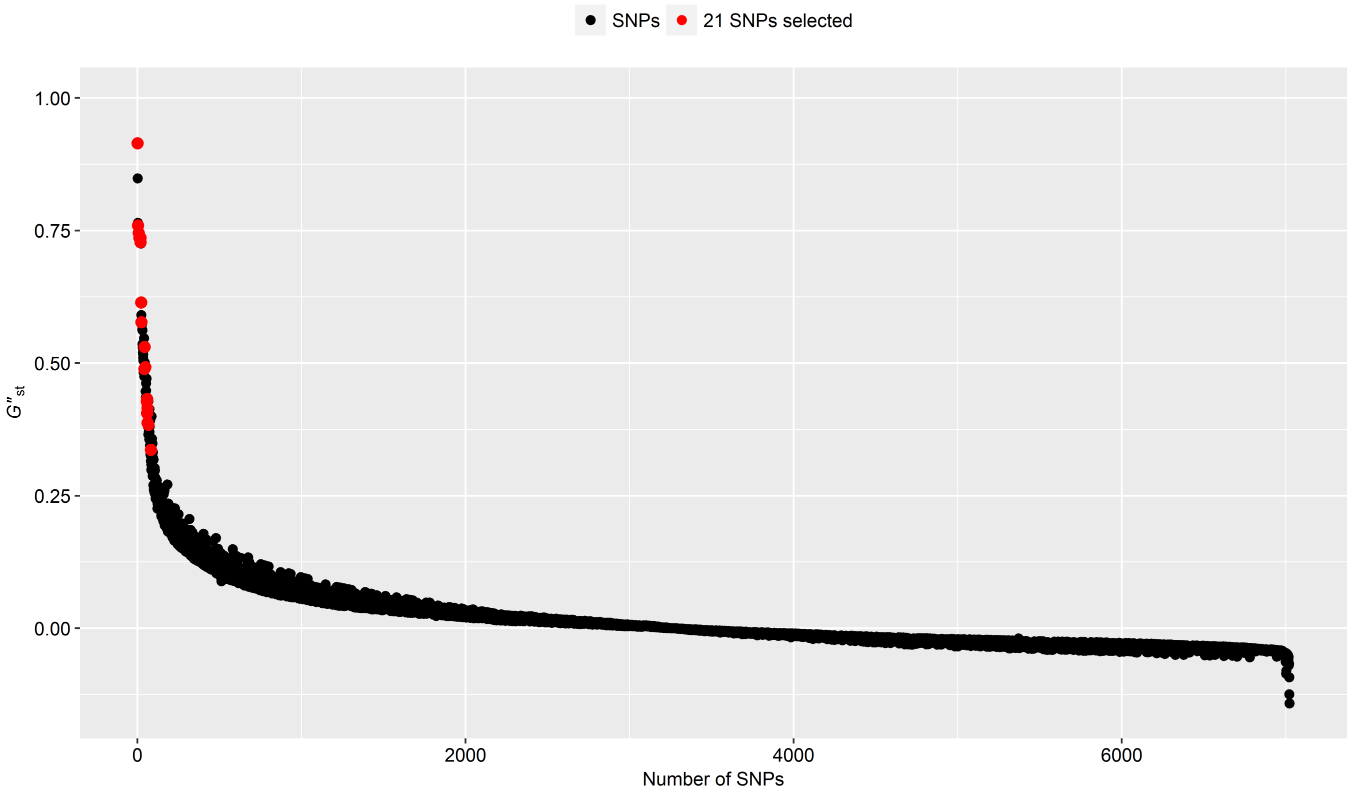

Figure S1C SNPs ranked by G''_{st} when all samples were grouped by Atlantic, Skagerrak or Mediterranean. Red points denote SNPs that were selected to compose the SNP panel.

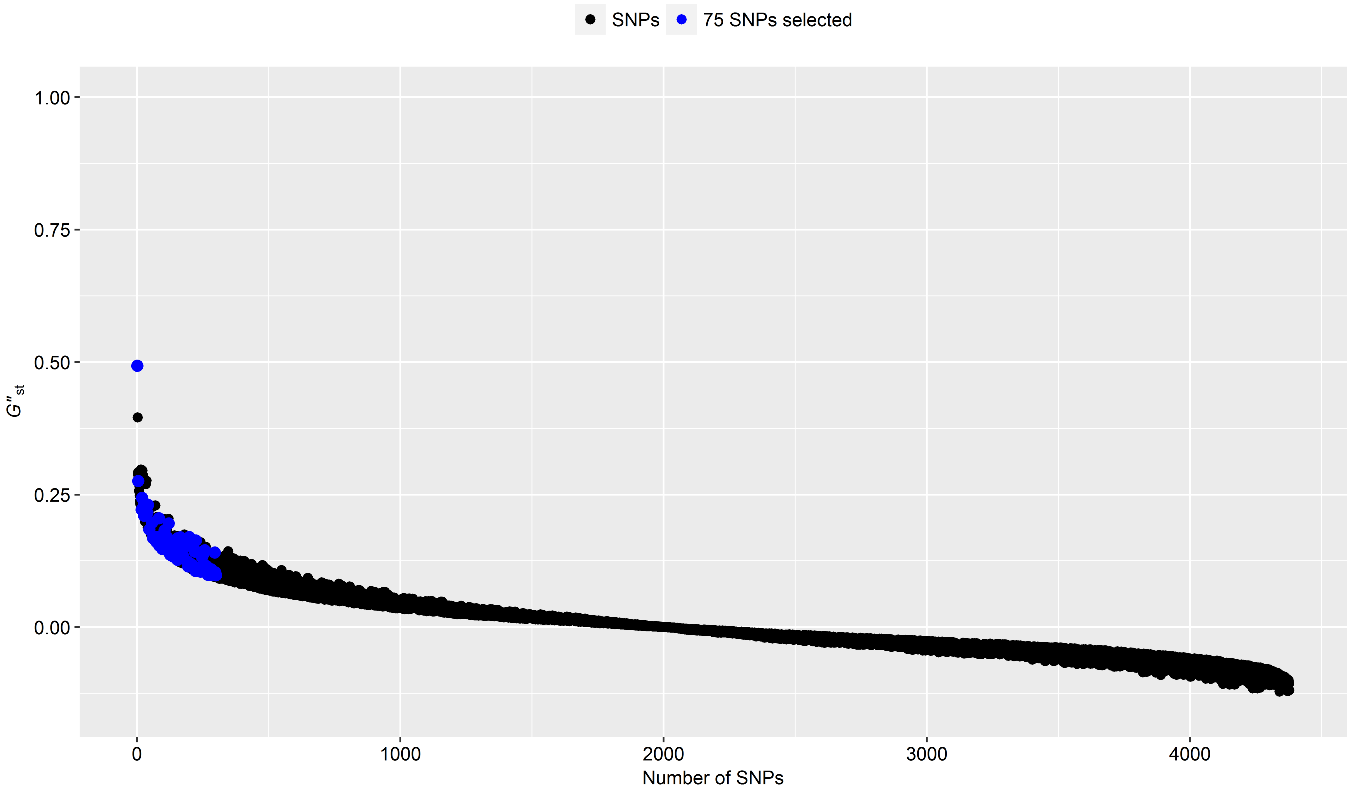

Figure S1D SNPs ranked by G''_{st} when Atlantic samples were grouped by geographic region (Table S1). Blue points denote SNPs that were selected to compose the SNP panel.
